# A novel microglia-targeting strategy based on nanoparticle-mediated delivery of miR-26a-5p for long-lasting analgesia in chronic pain

**DOI:** 10.1186/s12951-024-02420-9

**Published:** 2024-03-23

**Authors:** Yitian Lu, Shuai Liu, Peng Wang, Xiangna Guo, Zaisheng Qin, Honghao Hou, Tao Tao

**Affiliations:** 1 Department of Anesthesiology, Zhujiang Hospital, Southern Medical University, Guangzhou, Guangdong People’s Republic of China; 2 Guangdong Provincial Key Laboratory of Construction and Detection in Tissue Engineering, School of Basic Medical Sciences, Southern Medical University, Guangzhou, Guangdong People’s Republic of China; 3 Department of Anesthesiology, Nanfang Hospital, Southern Medical University, Guangzhou, Guangdong People’s Republic of China; 4 Department of Anesthesiology, Central People’s Hospital of Zhanjiang, Zhanjiang, Guangdong China; 5 Neuroscience Research Institute and Department of Neurobiology, School of Basic Medical Sciences, Key Laboratory for Neuroscience, Ministry of Education/National Health Commission, National Health Commission and State Key Laboratory of Natural and Biomimetic Drugs, Peking University, Beijing, China

**Keywords:** MG1, Nanoparticles, Chronic pain, Microglia, Analgesia

## Abstract

Accumulating evidence supports the notion that microglia play versatile roles in different chronic pain conditions. However, therapeutic strategies of chronic pain by targeting microglia remain largely overlooked. This study seeks to develop a miRNA-loaded nano-delivery system by targeting microglia, which could provide a decent and long-lasting analgesia for chronic pain. Surface aminated mesoporous silica nanoparticles were adopted to load miR-26a-5p, a potent analgesic miRNA, by electrostatic adsorption, which can avoid miR-26a-5p is rapidly released and degraded. Then, targeting peptide MG1 was modified on the surface of aminated mesoporous silica particles for microglia targeting. In peripheral nerve injury induced neuropathic pain model, a satisfactory anti-allodynia effect with about 6 weeks pain-relief duration were achieved through targeting microglia strategy, which decreased microglia activation and inflammation by Wnt5a, a non-canonical Wnt pathway. In inflammatory pain and chemotherapy induced peripheral neuropathic pain, microglia targeting strategy also exhibited more efficient analgesia and longer pain-relief duration than others. Overall, we developed a microglia-targeting nano-delivery system, which facilitates precisely miR-26a-5p delivery to enhance analgesic effect and duration for several chronic pain conditions.

**Graphical Abstract:**

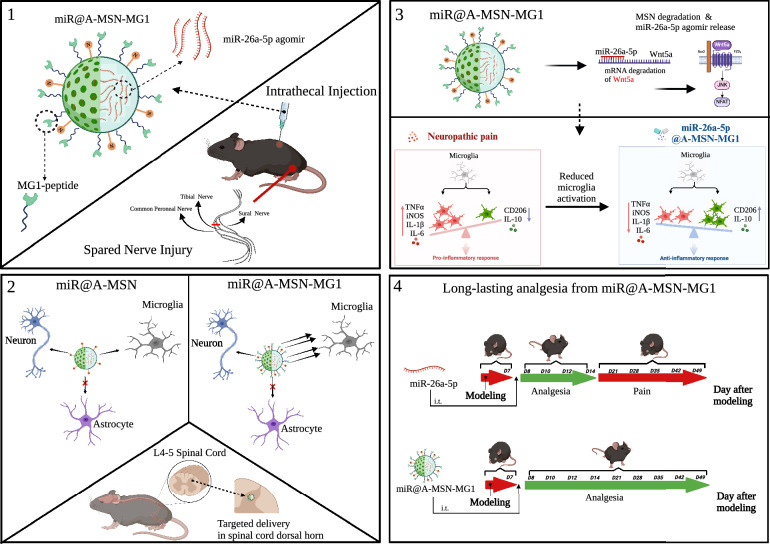

**Supplementary Information:**

The online version contains supplementary material available at 10.1186/s12951-024-02420-9.

## Introduction

Chronic pain is a prominent public health issue with a prevalence rate of 11–40% globally, impairing the social, psychological, and physical aspects of individuals [1] and producing significant disability and a considerable disease burden worldwide, as reported in The Global Burden of Disease Study 2017 and 2019 [2–4]. Based on the International Association for the Study of Pain (IASP), chronic pain is divided into three main classifications: nociceptive (involving tissue or potential tissue damage), neuropathic (involving nervous system injury), and nociplastic (persistent pain with no evidence of tissue or nerve injury) [5]. Currently, pharmacological treatment, including non-steroidal anti-inflammatory drugs (NSAIDs), central nervous system-acting drugs and opioids, is still an important part for chronic pain management [1, 6–8]. However, few patients get sufficient pain relief from current analgesics. Additionally, almost all these drugs could build up drug tolerance in patients, subsequently requiring larger doses to achieve same effect, which inevitably leads to a series of problems, including drug abuse, addiction, and intolerable drug-related side effects [1, 6, 8, 9]. Therefore, developing innovative therapeutic strategies to overcome or minimize above problems in pharmacological treatment is a pressing need for chronic pain management.

Recent advances in nucleic acid production and cellular delivery have attracted extensive research efforts to develop small RNA therapeutics in the central nervous system [10]. Compared with traditional drugs, small RNA therapeutics possess several advantages, including long-lasting effects, lower risk of drug resistance or tolerance, and cost-effectiveness in manufacture [11]. MicroRNAs (miRs), a kind of small endogenous non-coding RNAs, play essential roles in chronic pain through the regulation of gene expression and exhibit promising potential for developing chronic pain therapeutics [12]. We recently found that miR-26a-5p could produce evident analgesic effects in neuropathic and inflammatory pain, respectively [13, 14]. However, the analgesic duration of miR-26a-5p mimic is less than 1 week, owing to the rapid half-life of microRNA degradation in vivo [12]. To this end, a sustained release and targeted delivery system is preferred. As a competitive strategy for drug loading and targeted delivery, nanoparticles based drug delivery systems have been proved as versatile and effective vehicles for various biomolecules including nucleic acid [10, 15, 16]. Among them, surface-modified mesoporous silica particles (MSN), a well-known drug delivery workhorse, was chosen for loading and targeted delivery of microRNA in this study due to its highly accessible surface areas, interpenetrating mesoporous structure, facile surface modifications, and well biocompatibility [16–18].

On the other hand, numerous studies have found that microRNA could influence microglia function in response to various stimuli and stress by regulating neuroinflammation [19, 20]. Both our and previous investigations have found that miR-26a-5p exhibits excellent anti-inflammatory effects in chronic pain [13, 14, 21]. Meanwhile, accumulating evidence found that microglia is an essential cell type in the molecular and physiological mechanism in chronic pain, which accounts for synaptic remodeling, connectivity, and network function in response to inflammatory stimuli [7, 22]. Therefore, a growing drug development efforts are focusing on microglia as therapeutic target for chronic pain management [22–24]. The MG1 peptide has been found to have a high affinity for M1 microglia, which has been demonstrated to guide nanoparticles into microglia cells and enhance the therapeutic effect in autism spectrum disorder and neuroinflammation [25, 26]. Hence, the MG1 peptide is a promising guide for nanoparticle delivery systems in central nervous system diseases. Thus, in the current study, we hypothesize that A-MSN-MG1 could deliver miR-26a-5p to regulate neuroinflammation in spinal cord microglia, providing analgesia in various chronic pain conditions.

Herein, to demonstrate enhanced efficacy and long-lasting analgesic performance, we propose a novel microglia-targeting strategy using nanoparticle-mediated miR-26a-5p delivery for chronic pain management. We demonstrated that A-MSN-MG1 delivery system exhibited excellent biocompatibility and significantly enhanced microglia enrichment. More importantly, miR-26a-5p, when delivered by A-MSN-MG1 system, could efficiently provide a pain-relief duration of 6–7 weeks, extending at least 2 weeks longer than non-modified non-modified MSN delivery. Furthermore, the delivery of miR-26a-5p via A-MSN-MG1 exhibited excellent anti-inflammatory effects and effectively reduced the microglial activation. Lastly, we also demonstrated that miR-26a-5p@A-MSN-MG1 could provide effective analgesia for inflammatory pain and chemotherapy-induced peripheral neuropathic pain. Taken together, our study provides a safe and efficient miR-based therapeutic strategy for several chronic pain conditions.

## Results and discussion

### Preparation and characterization of MSN based miR-26a-5p targeted delivery systems

**Fig. 1:**
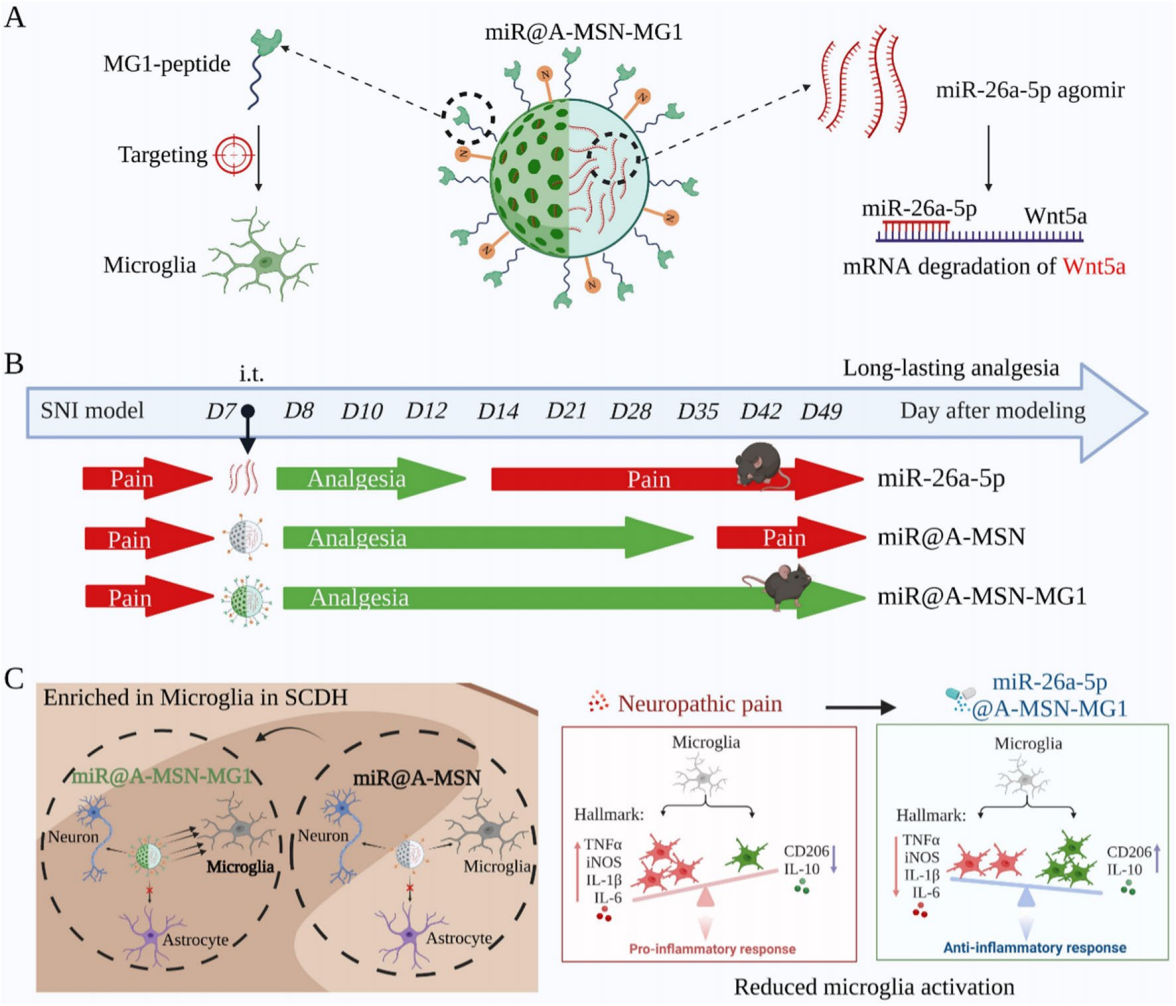
Schematic of miR-26a-5p nanoparticle targeted delivery system and long-lasting analgesia through anti-inflammation. **A** Mesoporous silica nanoparticles modified with MG1-targeting peptide and loaded with miR-26a-5p which targeted Wnt5a. **B** miR-26a-5p nanoparticle targeted delivery system provides long-lasting analgesia. **C** MG1 modification increases nanoparticle enrichment in microglia and efficiently regulate neuroinflammation. (A: Ethanol APTES, MSN: Mesoporous silica nanoparticle)

**Fig. 2:**
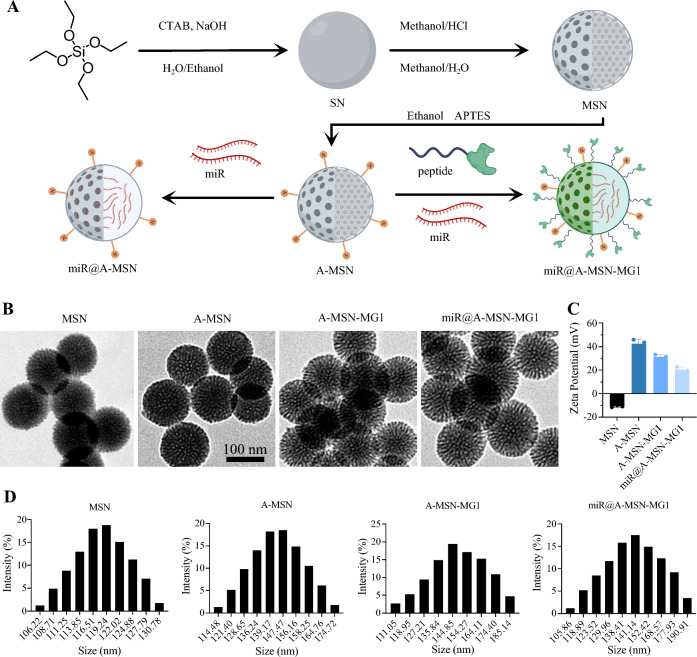
Preparation of nanoparticles and their particle size and potential characterization. **A** Schematic diagram of the preparation of mesoporous silica nanoparticles (MSN), aminated mesoporous silica nanoparticles (A-MSN), miR-loaded mesoporous silica nanoparticles (miR@A-MSN), and miR-loaded mesoporous silica nanoparticles with modified targeting peptides (miR@A-MSN-MG1). **B** Transmission electron microscope (TEM) images of MSN, A-MSN, A-MSN-MG1 and miR@A-MSN-MG1. **C** Zeta potential of MSN, A-MSN, A-MSN-MG1 and miR@A-MSN-MG1. **D** Particle size distribution of MSN, A-MSN, A-MSN-MG1 and miR@A-MSN-MG1

In contrast to other materials widely used in drug delivery such as liposomes and polymeric nanoparticles [27–29], MSNs are resistant and allow sustained release over time due to their well stability and controllable-release capacity [30, 31]. Thus, MSNs are potentially good choices among various nanocarrier for solving the problems of sustained drug release and targeted delivery [32, 33]. For better delivery of miR-26a-5p to microglia, as shown in Figs. 1A and 2A, herein we prepared four different types of MSN: (i) bare ones, with silanol groups on the surface and negative ζ potentials, denoted as MSN, (ii) surface amino-functionalized mesoporous silica nanoparticles A-MSN, obtained by co-condensation between tetraethylorthosilicate (TEOS) and (3-aminopropyl) triethoxysilane (APTES), which present amino groups and possess positive ζ potentials for better anchoring of therapeutic miRNAs, (iii) miR@A-MSN, prepared by loading miR-26a-5p into A-MSN via multiple non-covalent interactions, including electrostatic and macromolecular entanglement (iv) miR@A-MSN-MG1, obtained by grafted targeted peptides on the surface of miR@A-MSN. To investigate the physical properties of the nanoparticles, we performed the following experiments by transmission electron microscopy (TEM), and dynamic light scattering (DLS) to characterize the various nanoparticle systems. The results showed that all four types of nanoparticles exhibit similar pore arrangements and textural properties with concentrated particle sizes ranging between 100 and 200 nm (Fig. 2B, D). The mean hydrodynamic sizes, as determined by DLS, of A-MSN_,_ miR@A-MSN, miR@A-MSN-MG1, were found to be increased to varying extents compared to bare MSN, ranging from about 120 nm to 150 nm, also suggesting the successful surface modification (Fig. 2D). Moreover, the results of the zeta potential test showed that after the surface of MSN was modified with amination, the potential increased from approximately − 10 mV to + 43 mV, indicating successful grafting of a large number of amino groups onto the surface of MSNs, and the dispersion stability of the nanoparticles in solution was improved. When the surface of A-MSN was modified with the targeting peptide MG1, its zeta potential started to decrease, indicating that part of the NH_2_ reacted with MG1, while the potential of the nanoparticles further decreased after loading miR-26a-5p, also indicating the successful loading of miR-26a-5p and its potential was around + 20 mV, which still had good dispersibility (Fig. 2C).

**Fig. 3:**
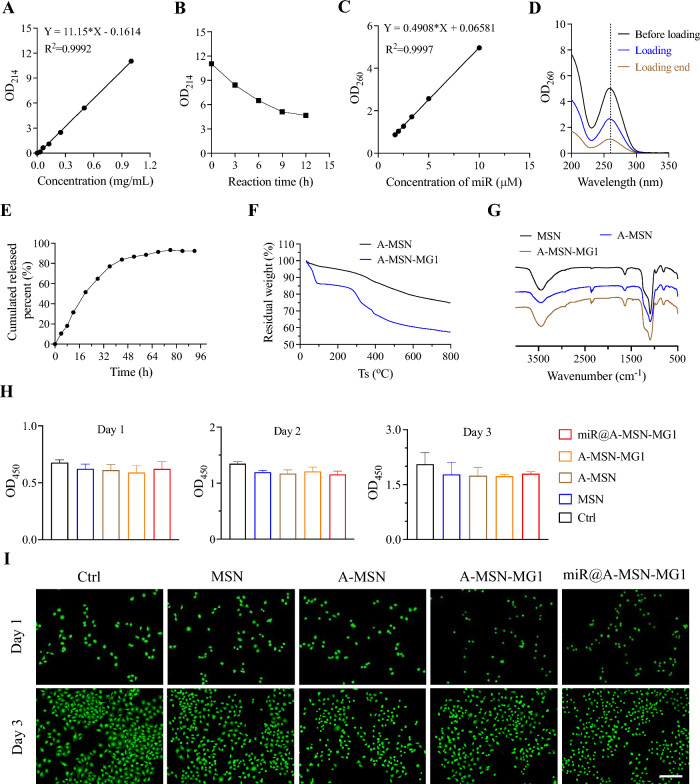
Physicochemical characterization and cytocompatibility testing of nanoparticles. **A** Standard curve for targeting peptide MG1 (absorbance—concentration). **B** Variation of targeting peptide MG1 concentration in solution with time of chemical grafting reaction. **C** Standard curve of miR (absorbance-concentration). **D** Concentration changes during the loading of miR into nanoparticles in solution. **E** In vitro simulation of miR release profile (release ratio–time). **F** Thermogravimetric analysis of A-MSN, A-MSN-MG1. **G** Fourier transform infrared (FTUR) spectra of MSN, A-MSN and A-MSN-MG1. **H** Toxicity test for cell proliferation viability of MSN, A-MSN, A-MSN-MG1 and miR@ A-MSN-MG1 (CCK-8). **I** Cytotoxicity experiments of MSN, A-MSN, A-MSN-MG1 and miR@ A-MSN-MG1 (live-dead staining)

To confirm and determine the successful drug loading and targeting peptide grafting, we used an ultramicro ultraviolet spectrophotometer to check the miR-26a-5p loading rate and MG1 grafting rate in MSN based miR-26a-5p targeted delivery systems. As shown in Fig. 3A, C, the absorbance peak values of both MG1 and miR-26a-5p solutions were found to be linear with respect to their concentrations, so we further determined the response rate of MG1 and the loading rate of miR-26a-5p by the standard curve of the MG1 and miR-26a-5p. As shown in Fig. 3A, B, the absorbance of MG1 in the solution decreased from 11.04 to 4.64 at the end of the reaction, with a response rate of 57.97%. Similarly, from Fig. 3C, D, it can be seen that the concentration of miR-26a-5p in the solution changed significantly before and after the reaction, and the loading rate was calculated to be 78.54%. The results of in vitro miR-26a-5p release assay also fully demonstrated the slow and sustained release behaves of the nano-delivery system, which showed a linear release within the first 48 h, followed by a faster release, and then a slower release between 48 and 96 h, and the final release rate could reach nearly 92%. In addition, considering the formulated forms of miRNAs (including miR-26a-5p) are easily degraded. Therefore, we performed a stability study. We measured the miRNA content in nanoparticles stored for 9 and 12 months (Additional file 1: Figure S1A). The results showed that the miRNA content in the nanoparticles remained almost unchanged between 9 and 12 months compared to freshly prepared nanoparticles, reflecting the good stability of our system. In addition, we also used artificial cerebrospinal fluid (ACSF) to simulate the in vivo environment and performed release kinetics experiments on nanomaterials stored for 12 months. The results showed that the release rates of nanoparticles in ACSF were almost the same for 0 and 12 months (Additional file 1: Fig. S1B). Then the thermogravimetric analysis (TGA) and Fourier transform infrared spectroscopy (FTIR) were performed to verify the successful surface modifications of nano-systems (Fig. 3F and 3G). From the TGA results in Fig. 3F, it is indicated that the weight reduction of MSN-NH_2_ was about 74% of that at room temperature when the temperature was increased to 800 ℃, indicating that the amino siloxane group was successfully modified to MSN. Meanwhile, the thermogravimetric curves of A-MSN-MG1 showed that the mass of the nanoparticles modified with MG1 decreased more with the increase of temperature (the final dry weight percentage was 57%), and the two curves remained approximate at the later stage, which also fully indicated that MG1 was successfully modified into A-MSN nanoparticles.

As observed in Fig. 3G, the characteristic peak of the hydroxyl group on the surface of MSNs (σ = 1081 ppm) was significantly reduced after chemical modification, indicating that the reaction with APTES occurred and consumed part of the hydroxyl group. New characteristic absorption peaks (σ = 2350 ppm) appeared in the FTIR spectra of A-MSN and A-MSN-MG1, which likely represent the characteristic absorption peaks of amino groups [31]. Also a new absorption peak appeared at σ = 1490 ppm, which is the characteristic absorption peak of the amide group, indicating the successful modification of the targeting peptide onto the MSN-NH_2_ nanoparticles. In accordance with the results of TEM and DLS measurements, the above results of thermogravimetric analysis and infrared spectroscopy further confirmed the effective chemical modification with amino groups and MG1 peptides and of the nanoparticles. The design and establishment of targeted nano-delivery system are expected to not only achieve targeting, but also endow the potential for prolonged release and therapeutic performance.

### In vitro biocompatibility of nano-delivery systems

Besides the above physicochemical properties, we further checked the in vitro cytocompatibility of nanoparticles delivery systems via cell proliferation toxicity assay and cell live-dead staining assay (Fig. 3H, I). We incubated human umbilical vein endothelial cells (HUVECs) with bare MSNs, A-MSN_,_ miR@A-MSN, miR@A-MSN-MG1 at the same concentrations in the culture medium until examination, alongside control groups receiving no treatment. As expected, from the results of the CCK-8 assay, the results showed that the absorbance of HUVECs cultured in nanoparticle-containing medium was not significantly different from that of the control group at 450 nm, indicating that the four types of nanoparticles no noticeable toxicity to cell proliferation. In addition, as seen in Fig. 3I, the live-dead staining results revealed robust cell growth with almost no dead cells observed. Also the cell density and morphology were not significantly different from the control group after cultured by the medium containing nanoparticles, with a significantly higher number of live cells compared to dead cells, suggesting the good cytocompatibility of nanoparticles. The results of in vitro biocompatibility of nano-delivery systems fully show that the surface modification with amino- functionality and MG1 graft did not decrease the inherent advantages of well biocompatibility for MSNs systems, while also imparting the capability to target microglia, indicating a great potential for long-lasting targeted release system [34, 35]. Furthermore, HE staining of the liver and kidneys from mice in different treatment groups revealed no apparent alterations or signs of inflammation following nanoparticle administration (Additional file 1: Fig. S2A). Additionally, we evaluated liver function markers (AST and ALT) and kidney function markers (BUN and Cr) (Additional file 1: Fig. S2B), and the results showed no significant changes, further supporting the safety profile of our nanoparticles.

### miR-26a-5p@A-MSN-MG1 delivery system produce long-lasting analgesia duration

Although increasing specific miRNAs has been accepted as a promising therapeutic strategy in chronic pain, current miRNA mimics cannot provide long-lasting or sustainable analgesia which may be partly due to the nature of miRNAs, e.g. their negative charge [39], short half-life in vivo [36, 37], and rapid degradation and inactivation by endogenous nucleases [38, 39]. Apart from that, the non-specificity of miRNA on different cell types also attenuates the effect in pain conditions [12].

**Fig. 4:**
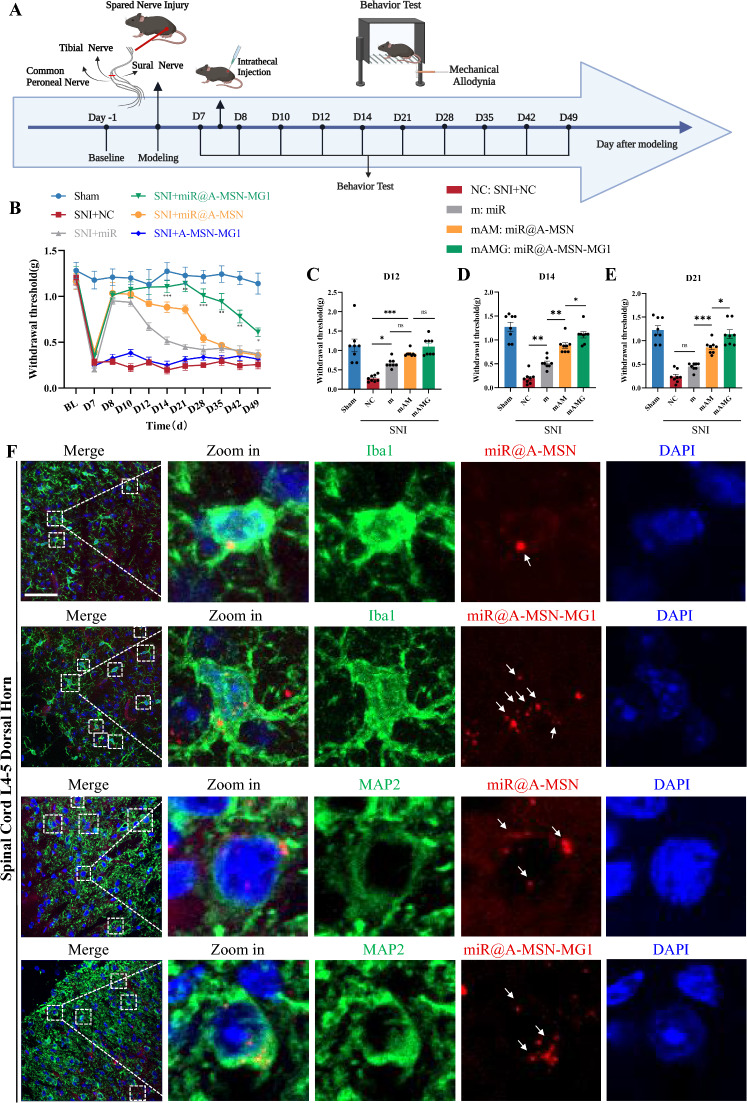
mir-26a-5p@A-MSN-MG1 delivery system produce long-lasting analgesia duration. **A** Experimental schematic plot for the establishment of the mouse model of spared nerve injury (SNI). **B** 50% paw withdraw threshold (PWT) of left hind paw of different treatment groups mice. At the day of 14, 21, 28, 35, 42 and 49, the paw withdrawal thresholds (WTs) of SNI + miR@A-MSN-MG1 group was significantly higher than those of SNI + miR group after single intrathecal injection at the 7th day (*p < 0.05, **p < 0.01, ***p < 0.001 compared with SNI + miR group by Two-way ANOVA followed by Tukey post hoc test, n = 8 in each group). **C-E** 50% paw withdraw threshold (PWT) of left hind paw of different treatment groups mice at day 12, 14 and 21. **F** Immunofluorescent study revealed that the enrichment of nanoparticles (Cy5 red) in microglia (IBA1 green) in the L4–5 spinal dorsal horn of SNI mice was significantly increased after MG1 targeting peptide modification but there was no significant change in neurons (MAP2 green). The blue spots are DAPI nuclear staining (Scale bar: 50 μm)

To address these challenges, we employed mesoporous silica nanoparticles (MSN) for miR-26a-5p mimic delivery and further modified the surface of MSN with a microglia-targeting peptide, MG1, to improve its duration and targetability in vivo. Subsequently, we established a spared nerve injury (SNI) mouse model to evaluate the pain-relieving effect adopting different delivery strategies. Our behavioral tests showed that miR-26a-5p@A-MSN-MG1 could provide a 42-days analgesia period after single administration, whereas miR-26a-5p@A**-**MSN or miR-26a-5p mimic only provide analgesic periods of 21 days and 5 days, respectively (Fig. 4B–E). These results suggested that mesoporous silica nanoparticles (MSN) could efficiently elongate the effect of miRNA mimic, which may be attributed to its notable stability and controllable-release capacity [30, 31]. More importantly, MG1-targeted modification further extend the analgesic duration to 42 days (Fig. 4B–E), which, to the best of our knowledge, is longer than any previously reported analgesic duration using nanoparticles or exosomes to deliver miRNA or small molecule (Table 1) [32, 40, 41]. Data of mechanical pain for D8, 10, 28, 35, 42 and 49 are shown in Additional file 1: Fig.S3.

**Table 1:** Analgesic efficacy of different delivery systems

Delivery system	Loaded molecules	Pain model	Analgesia duration	Refs.
hPMSCs-exosomes	miR-26a-5p	Neuropathic pain	28 days	[13]
MSN	Δ9-THC and ARA290	Neuropathic pain	28 days	[32]
miR@A-MSN-MG1	miR-26a-5p	Neuropathic pain	42 days	This work
Microcapsule	QX-314	Inflammatory pain	12 days	[41]
Huc-MSCs-exosomes	miR-146a-5p	Inflammatory pain	7 days	[40]
None	miR-26a-5p	Inflammatory pain	5 days	[14]
miR@A-MSN-MG1	miR-26a-5p	Inflammatory pain	at least 14 days	This work

We have also conducted open-field and rotarod experiments at corresponding time points (Additional file 1: Fig. S4). The results indicate no significant differences in the mice's motor function, excluded that pain-like behavior obtained are due to compounding factors.

To further observe the targeting effect of MG1 modification in vivo, we labeled the nanoparticles with Cy5 and conducted immunofluorescence staining on the L4-5 segments of the mouse spinal cord. We observed that following intrathecal injection of miR-26a-5p@A-MSN, red fluorescence co-localization was noted in both microglia (IBA1^+^) and neurons (MAP2^+^) (Fig. 4F), but almost no co-localization in astrocytes (GFAP^+^) (Additional file 1: Fig. S2A). After modification with the targeting peptide MG1, an increased presence of Cy5-labeled nanoparticles was observed in microglia, with a greater number of microglia colocalizing with these nanoparticles (Fig. 4F, Additional file 1: Fig. S2B). We have also captured images of the contralateral side, revealing that there is more Cy5 red fluorescence on the ipsilateral side, indicating a higher concentration of miR@A-MSN-MG1 (Additional file 1: Fig. S5). This may be attributed to the greater activation of M1-type microglial cells on the ipsilateral side compared to the contralateral side, as clearly observed in the figure. Compared with microglia, MG1 modification did not changed the colocalization of nanoparticles with neurons and astrocytes (Additional file 1: Fig. S6A). These results indicated that MG1 modification could significantly facilitate more nanoparticles targeting microglia, and thus result in the extended analgesic duration after modification. Regrettably, the nanoparticles could still be observed in neuron after MG1 modification, suggesting a need for further efforts to improve the specificity and exclusivity of the nano-delivery system for microglia. MG1 peptide, a homing peptide that specifically recognizes M1 microglia, was identified and isolated by phage display technology [42]. Previous studies showed that BV2-M1 cells showed significantly higher uptake of MG1-modified nanoparticles than mouse brain vascular endothelial cells (bEnd.3), neuron-like PC12 cells, and rat brain astrocytes (CTX) [26]. Although miR@A-MSN-MG1 could not avoid uptake by neurons or other cells, it significantly enhanced enrichment in microglia, and the MG1 peptide still exhibited excellent targeting effects on microglia under inflammatory conditions [26].

We also confirmed the presence of nanoparticle accumulation in the DRG (Additional file 1: Fig. S7). However, our primary therapeutic target is the abundant activation of microglial cells in the spinal cord following neuropathic pain. The MG1 targeting peptide significantly increases the enrichment of nanoparticles in spinal microglial cells, enhancing our therapeutic strategy's specificity.

Moreover, in the current study, although nanoparticles have the ability to cross the blood–brain barrier [43], we adopted intrathecal injection as administration route instead of oral, intravenous or intraperitoneal route. The main reason is that intrathecal injection enables the miRNA mimic to act directly on spinal cord by delivering the nanoparticles to the subarachnoid space. Furthermore, intrathecal injection is a common method to reduce medicine consumption and minimize systemic medicine effects in pain management [44]. Therefore, in the current study, we utilized intrathecal injection to achieve optimal therapeutic effects with a lower dosage and minimal side effects [45].

### miR-26a-5p@A-MSN-MG1 delivery system contribute to the prolonged suppression of microglia activation and neuroinflammation

The role of microglia in the onset, progression, and maintenance in chronic pain is well established [23, 46]. A core feature of microglia adaptation to peripheral nerve injury is their activation, leading to persistent neuroinflammation [47]. Therefore, modulating microglia-associated inflammation has been considered as an effective strategy for pain management [22]. In current study, we further evaluated the effect of different delivery strategies on microglia activation and its associated inflammation.

**Fig. 5:**
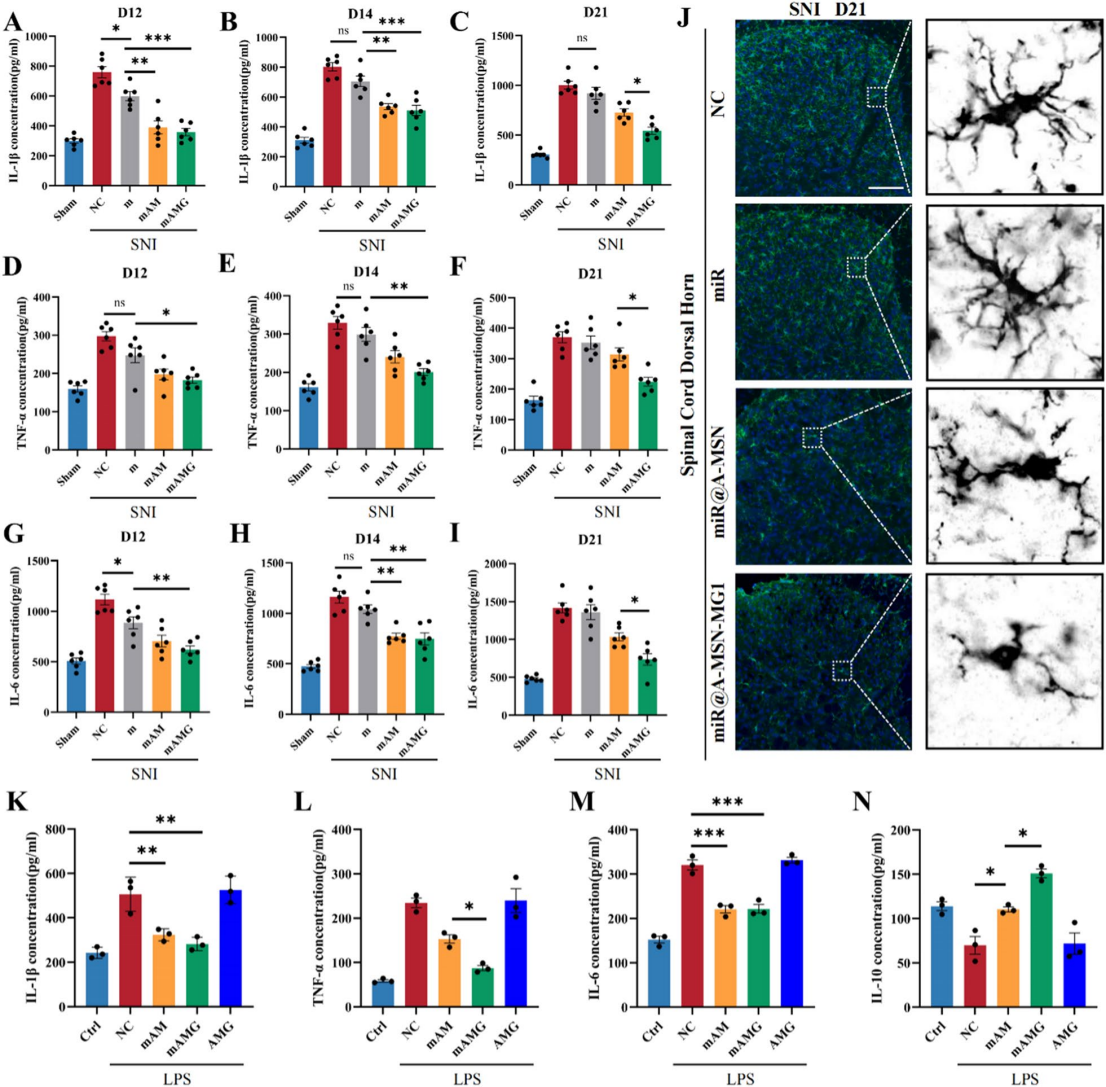
miR-26a-5p@A-MSN-MG1 delivery system contribute to the prolonged suppression of microglia activation and neuroinflammation. **A–I** Protein levels of IL-1β (**A–C**), TNF-α (**D–F**), and IL-6 (**G–I**), in the L4–L5 dorsal spinal cord of mice were tested by ELISA at postoperative day 12, 14 and 21. N = 6 mice for each group. Data are represented as mean ± sem.** J** Immunofluorescent study revealed IBA1-positive microglia (green) in the spinal dorsal horn of different treatment groups mice at day 21 (Scale bar: 100 μm). The blue spots are DAPI nuclear staining. **K-N** The protein levels of IL-1β (**K**), TNF-α (**L**), IL-6 (**M**), IL-10 (**N**) in BV2 cell culture supernatant were tested by ELISA

We firstly evaluated the anti-inflammatory effect of different delivery strategies in the L4-5 segment of spinal cord at POD 12, 14 and 21 after SNI. All three strategies to deliver miR-26a-5p mimic significantly attenuated the level of IL-1β and IL-6 (Fig. 5A, G). Consistent with behavioral results of mechanical allodynia, miR@A-MSN and miR@A-MSN-MG1 exhibited a prolonged anti-inflammatory effect untill POD 14 and 21, respectively (Fig. 5A–I). Next, we observed the long-term effect of MG1 modification on microglia activation in the dorsal horn of spinal cord, the L4-5 segment of mice spinal cord were sampled at POD 21, a timepoint that clearly distinguishes the analgesic and anti-inflammatory effects of A-MSN and A-MSN-MG1 delivery strategies. The immunofluorescent staining results showed that the effect of miR-26a-5p in inhibiting microglial activation showed no statistical difference compared to the NC group at POD 21 (Fig. 5J and Additional file 1: Fig. S3). Compared with other group, the number of activated microglia in the miR@A-MSN-MG1 group is the lowest (Additional file 1: Fig. S8). These results indicated that the longer analgesic duration of A-MSN or A-MSN-MG1 delivery system is mainly due to the sustained effect on microglia activation and anti-inflammation. We also noted that the decreases in IL-1β, TNF-α, and IL-6 levels did not occur in parallel at each timepoint, which may be related to the distinct roles of these inflammatory cytokines in the progression of chronic pain [48].

Subsequently, we further evaluated the anti-inflammatory effect of MG1 modification in vitro. BV2 cells, cultured and stimulated with LPS (1 μg/mL) for 24 h. Meanwhile, the miR-26a-5p@A-MSN or miR-26a-5p@A-MSN-MG1 were added to the culture medium to reach the 100 nM concentration. Compared with other groups stimulated with LPS, miR-26a-5p@A-MSN or miR-26a-5p@A-MSN-MG1 both significantly decreased the level of IL-1β, TNF-α and IL-6, and increased the level of IL-10 in culture supernatant (Fig. 5K–N). These in vitro results suggest that both MSN and MG1 modified MSN strategies could exert excellent anti-inflammatory effects. Of note, miR-26a-5p@A-MSN-MG1 exhibits a more evident effect in TNF-α and IL-10 level (Fig. 5L, N) compared with miR@A-MSN in vitro, this further indicates that targeting microglia is a better approach to combat inflammation.

In past decades, accumulating studies have provided evidence for the important role of microglia in allodynia and central sensitization of chronic pain and have shed light on the development of therapeutic strategy [22]. Previous studies adopted minocycline (an inhibitor of microglia activation) and chemogenetic manipulation of microglia are both effective targeting strategies in rodent chronic pain model through inhibiting microglia activation and inflammation [48]. These studies and our targeting strategy demonstrated that target microglia to regulate inflammation is a potent approach for chronic pain management. Moreover, MG1 modified strategies are unlikely to cause antibiotic-related adverse events resulting from minocycline and are more feasible to perform compared with chemogenetic manipulation.

### miR-26a-5p@A-MSN-MG1 delivery system maintains a prolonged regulation on Wnt5a signaling pathway

**Fig. 6:**
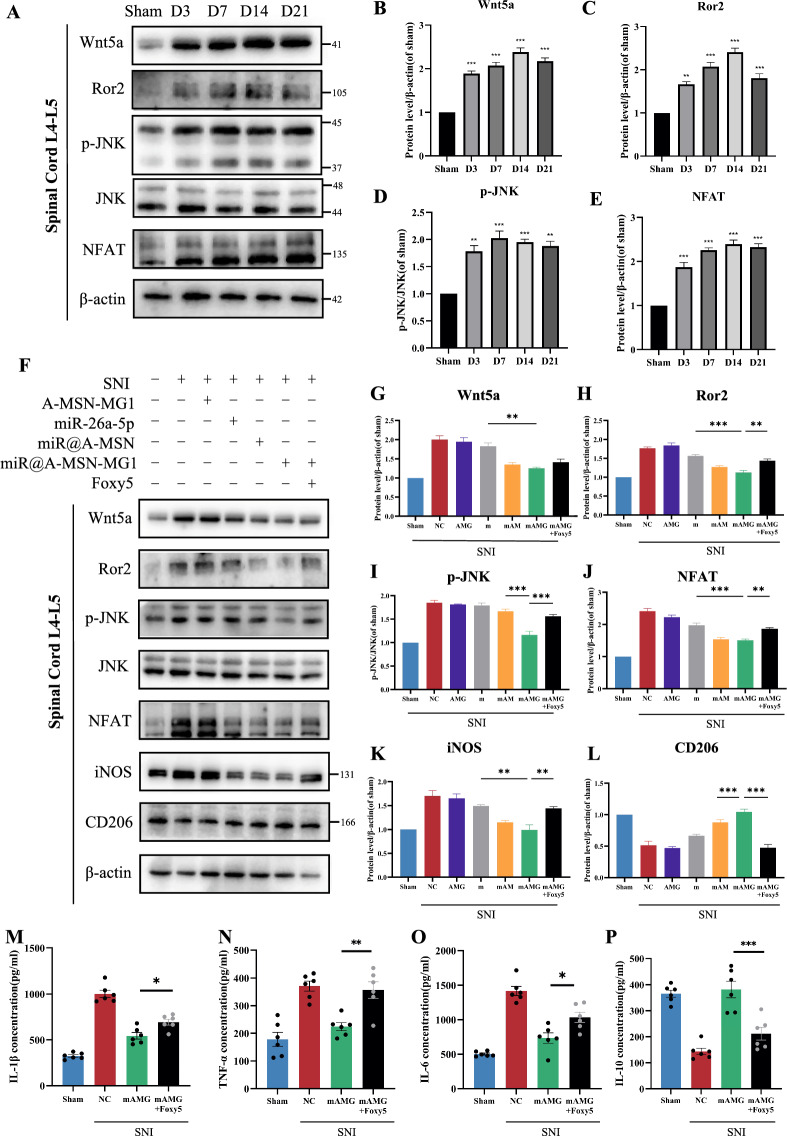
miR-26a-5p@A-MSN-MG1 delivery system maintains a prolonged regulation on Wnt5a signaling pathway. **A–E** Western blot was used to assess the level of Wnt5a (**B**), Ror2 (**C**), p-JNK (**D**) and NFAT (**E**) at day 3, 7, 14, and 21 after SNI modeling. Data are expressed as fold change compared to the sham group. **F–L** Western blot was used to assess the level of Wnt5a (**G**), Ror2 (**H**), p-JNK (**I**) and NFAT (**J**), iNOS (**K**) and CD206 (**L**) in different treatment groups. Representative immunoblots and quantification showing Foxy-5 reversed the effect of miR@A-MSN-MG1. Data are expressed as fold change compared to the sham group. **M–P** Protein levels of IL-1β (**M**), TNF-α (**N**), IL-6 (**O**) and IL-10 (**P**), in the L4–L5 dorsal spinal cord of mice after Foxy-5 reverse were tested by ELISA at day 21. N = 6 mice for each group. Data are represented as mean ± sem. *p < 0.05, **p < 0.01, ***p < 0.001

Wnt5a, a member of non-classical Wnt signaling pathway, is a promising target for cancers, inflammatory diseases, and chronic pain [49–51]. Our previous research found that miR-26a-5p targets Wnt5a to produce analgesia through regulating neuroinflammation. On this basis, we observed the expression level of Wnt5a signaling molecules at different timepoints after SNI. We found that Wnt5a and its membrane receptor ROR2 were significantly elevated at POD 3,7,14,21, with the highest expression at POD 14(Fig. 6A–C, similar expression pattern could be found in p-JNK and the downstream transcription factor NFAT (Fig. 6A, D, E). These results demonstrated that Wnt5a signaling molecules, including Wnt5a, Ror2, p-JNK and NFAT, continue to elevate after SNI till POD 21. This also suggests that Wnt5a/Ror2-mediated non-classical Wnt signaling is an important signaling pathway in the progression of SNI-induced neuropathic pain towards chronicity, which continues to be activated and plays an important role for a longer period of time after the onset of pain [52], and thus Wnt5a/Ror2 signaling may be an important therapeutic target for chronic pain.

Next, we examined the expression of Wnt5a, ROR2, p-JNK and NFAT at POD 21 after SNI, employing different miR-26a-5p delivery strategies through intrathecal administration. Consistent with above results, all three miR-26a-5p delivery strategies could significantly decrease the protein expression of Wnt5a, ROR2, p-JNK and NFAT. miR-26a-5p**@**A-MSN-MG1 is the most effective deliver strategy in regulating molecules of Wnt5a signaling pathway (Fig. 6G–I). We also observed that miR-26a-5p decreased iNOS and increased CD206 (hallmark molecules of M1 and M2 microglia respectively) expression respectively which indicated that miR-26a-5p could influence the equilibrium of M1/M2 microglia and thus regulate microglia activation and inflammatory cytokines releasing. Furthermore, we also demonstrated the effect of miR-26a-5p on Wnt5a signaling pathway and microglia function could be reversed by Foxy5, a Wnt5a mimetic peptide, in miR-26a-5p@A-MSN-MG1 deliver system (Fig. 6G–L). These results further confirmed that A-MSN-MG1 could prolong the effect of miR-26a-5p on Wnt5a signaling pathway to regulate microglia function at least to POD 21 after SNI.

Wnt5a is a critical signaling molecule in physiological and pathological conditions, including embryonic development, tumors, inflammatory diseases, and neurodegeneration [53–55]. Previous research found that Wnt5a expression is responsible for sustained inflammation and macrophage activation through autocrine and paracrine signaling [56]. Our results also found that Wnt5a maintains an evident increasing expression pattern after SNI till POD 21 (Fig. 6A, B). Corresponding to Wnt5a expression level, the activation of microglia remained significantly elevated, accompanied by a marked upregulation of inflammatory cytokine expression (Fig. 5A–J). ROR2 and NFAT are both key molecules in Wnt5a signaling pathway to regulate inflammation and microglia function [54, 57]. Furthermore, recent studies showed that NFAT (Nuclear Factor of Activated T-cells) not just mediated T cells function, but contribute to innate immunity, including employed by myeloid cells during early immune response to pathogens and/or tissue lesion, and promote inflammation [58, 59]. In a mouse model of Alzheimer's disease, it has been demonstrated that inhibiting NFAT can attenuate microglial activation and subsequent inflammation [60]. Additionally, triggered NFAT-mediated transcription could amplify the pro-inflammatory responses of microglia [61]. Our results demonstrated that targeting microglia Wnt5a is a feasible strategy to regulate the ROR2 and NFAT expression, which are important molecules in Wnt5a signaling pathway to induce neuroinflammation and microglia activation. What’s more, our strategy to deliver miR-26a-5p provides a sustainable miRNA release and improve the cell-specificity, which contributes to the long-term inhibition on Wnt5a signaling induced neuroinflammation and microglia activation.

### miR-26a-5p@A-MSN-MG1 delivery system provides sustained relief from inflammatory pain and chemotherapy-induced peripheral neuropathic (CIPN) pain

Accumulating evidence suggests that Wnt5a is a crucial target in various types of chronic pain. Its involvement has been observed in EAE-induced chronic pain [62], gp120-induced mechanical abnormal pain [63], and diabetic neuropathic pain [64]. Blocking Wnt5a reduces the release of pro-inflammatory factors and decreases neuroinflammation while leading to a reduction in pain-related behaviors [51, 65–67]. We therefore adopted complete Freund's adjuvant (CFA)-induced inflammatory pain and chemotherapy-induced neuropathic pain mouse models to further evaluate the effect of A-MSN-MG1 delivery of miR-26a-5p in these two common chronic pain conditions in the clinic. Both conditions are closely associated with microglia activation and the mediated neuroinflammation [40, 47, 68, 69].

**Fig. 7:**
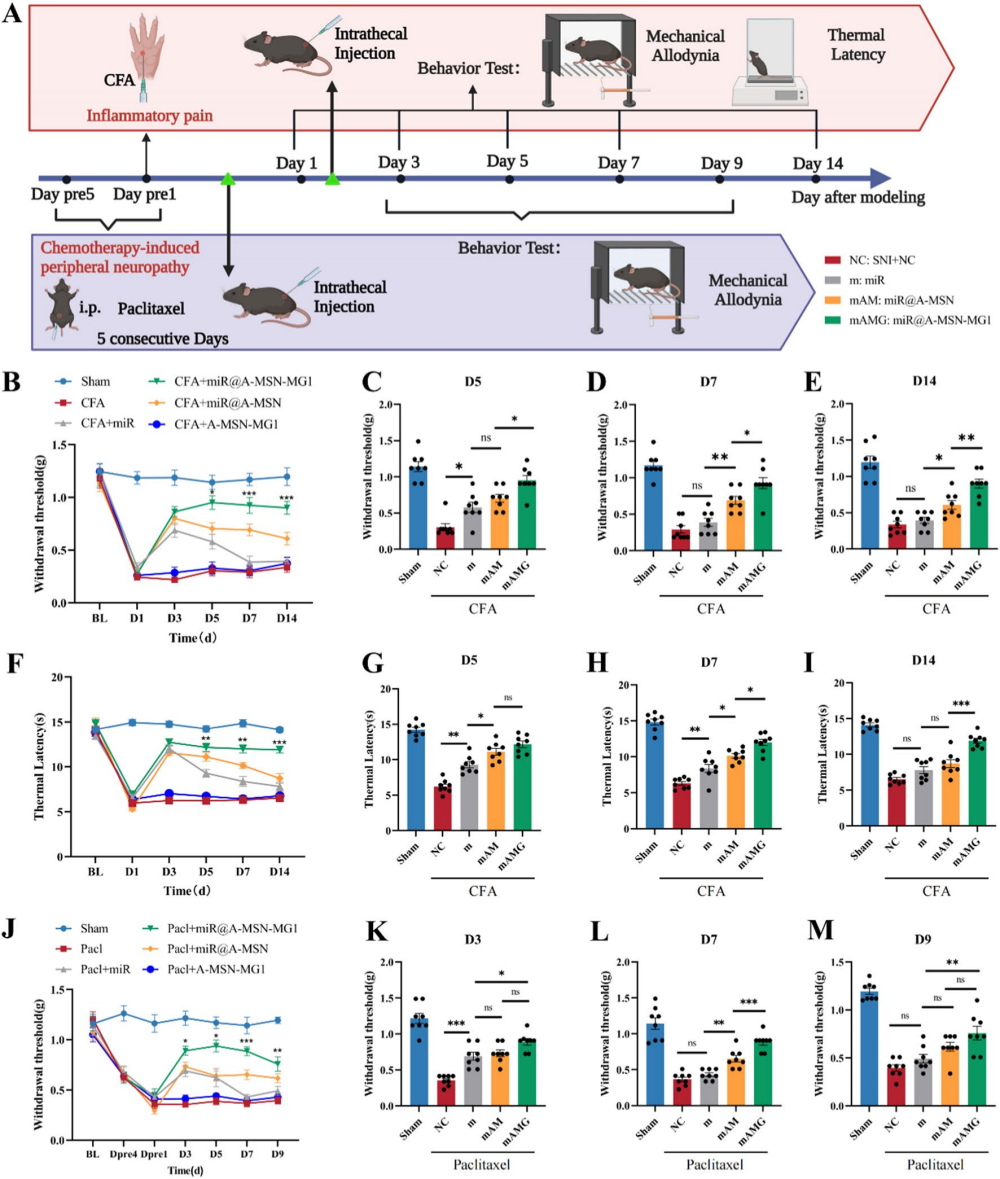
miR-26a-5p@A-MSN-MG1 delivery system provides sustained relief from inflammatory pain and Chemotherapy-induced peripheral neuropathic (CIPN) pain. **A** Experimental schematic plot for the establishment of the mouse model of inflammatory pain and chemotherapy-induced peripheral neuropathy. **B** 50% paw withdraw threshold (PWT) of left hind paw of different treatment inflammatory pain mice groups. At the day of 5, 7 and 14, the paw withdrawal thresholds (WTs) of CFA + miR@A-MSN-MG1 group was significantly higher than those of CFA + miR group. (*p < 0.05, **p < 0.01, ***p < 0.001 compared with SNI + miR group by Two-way ANOVA followed by Tukey post hoc test, n = 8 in each group). **C–E** 50% paw withdraw threshold (PWT) of left hind paw of different treatment groups of inflammatory pain mice at day 5, 7 and 14. **F–I** Thermal latency(s) of left hind paw of mice in different treatment inflammatory pain mice groups. **J–M** 50% paw withdraw threshold (PWT) of left hind paw of different treatment CIPN mice groups. Data are represented as mean ± sem

In the inflammatory pain model, we found that directly delivered miR-26a-5p significantly increased the mechanical threshold and thermal latency at POD 3 and POD 5 compared with NC group (Fig. 7B, F, C, G and Additional file 1: Fig. S9B). Compared with directly administered miR-26a-5p, A-MSN and A-MSN-MG1 delivery systems provided more evident analgesic effect at POD 7 and POD14 (Fig. 7B–I). Moreover, miR-26a-5p@A-MSN-MG1 exhibited the most evident improvement in mechanical threshold and thermal latency at POD 7 and 14 compared with other groups. These results suggested that A-MSN-MG1 delivery system could also provide the longest effective pain-relieving period in inflammatory pain among all three delivery strategies. This effect may also be related to the strategy of miR@A-MSN-MG1 targeting microglia to attenuate neuroinflammation. Data of mechanical and thermal pain for D1 and D3 are shown in Additional file 1: Fig. S9.

Then, we administered the paclitaxel intraperitoneally for 5 consecutive days to mimic CIPN pain and performed von Frey tests at POD 3, 5, 7, 9 (Fig. 7A). The analgesic effect and pain-relieving duration of miR-26a-5p, miR-26a-5p@A-MSN and miR-26a-5p@ A-MSN-MG1 showed a similar trend with neuropathic and inflammatory pain (Fig. 7J–M). miR-26a-5p alone just provides evident pain-relief until POD 3. Although miR-26a-5p@A-MSN and miR-26a-5p@A-MSN-MG1 could maintain significant analgesia till POD 9, there is no statistical significance between miR-26a-5p and miR-26a-5p@A-MSN (Fig. 7J), and between miR-26a-5p@MSN and miR-26a-5p @A-MSN-MG1 at POD 9 (Fig. 7M). Data of mechanical pain for pre4,1 and D5 are shown in Additional file 1: Fig. S10. These results indicate that miR-26a-5p@A-MSN-MG1 could still provide the most evident and longest pain-relief in chemotherapy-induced neuropathic pain. However, with the development of CIPN pain, miR-26a-5p@A-MSN-MG1 may not maintain an equivalent pain-relief duration as it does in neuropathic and inflammatory pain. This may attribute to the non-specific tissue damage and cell toxicity caused by chemotherapeutics. miR@A-MSN-MG1 could significantly alleviate the aseptic inflammation caused by this injury in the early stage by targeting microglia. However, in the advanced stage of CIPN pain, other mechanisms, including altered ion channel activity, mitochondrial dysfunction and myelin damage, are involved and make the situation so complicated that microglia-targeting strategy cannot achieve the anticipated effect [70] [71].

## Conclusion

In this study, we develop a facile and effective targeted nano-delivery system that can provide a long-lasting pain relief duration for several chronic pain conditions by loading miR-26a-5p. By modifying the targeting peptide MG1 on its surface, the enrichment of nanoparticles was significantly increased in microglia. MG1-modified MSNs-miR delivery system significantly enhanced the analgesic effect and extended the duration of miR-26a-5p analgesia in SNI mice model to 6–7 weeks, while exhibiting a similar effect and duration trend for inflammatory and CIPN pain. In summary, enhancing the precision of molecular and cellular targeting of MG1-MSNs-miR delivery system to achieve better analgesic effect is of great significance for the clinical development of miRNA analgesic drugs. Overall, our amino-modified MG1-MSNs miRNA delivery system provides a new, powerful strategy for the management of various chronic pain conditions.

## Experimental section

### Materials

Tetraethyl orthosilicate (TEOS), sodium hydroxide (NaOH), ethanol, cetyltrimethylammonium bromide (CTAB), methanol, hydrochloric acid (HCl), aminopropyltriethoxysilane (APTES), *N*, *N*-dimethylformamide (DMF), O-benzotriazole-tetramethyluronium hexafluorophosphate (HBTU), *N*,*N*-diisopropylethylamine (DIEA), targeting peptide (MG1, CHHSSSAR), miR-26a-5p.

### Preparation of mesoporous silica nanoparticles (MSN)

10.0 g of anhydrous ethanol, 90.0 g of deionized water and 0.05 g of NaOH were added into a 250 mL round bottom flask and stirred thoroughly at 40 ℃. Then 0.2 g of CTAB was added and stirred for 10 min. TEOS was added drop by drop under continuous stirring and stirred for 10 h. The sample was transferred to a 50 mL centrifuge tube and centrifuged at 4000 rpm for 10 min. The supernatant was discarded and washed with 10 mL of ethanol solution (2 mL ethanol/8 mL water), and the supernatant was discarded by centrifugation and repeated three times. The obtained precipitate was dried in a vacuum drying oven (50 ℃) overnight to obtain mesoporous silica nanoparticles (MSN).

### Preparation of amino-functionalized mesoporous silica nanoparticles (MSN-NH_2_)

Take 50 mg of the mesoporous silica sample prepared in the above steps in a beaker, add 8 mL of absolute ethanol, ultrasonically and stir to disperse evenly. Add 0.2 mL of APTES to 7 mL of ethanol, sonicate for 1 h to make it fully mixed. Mix the two mixtures obtained above, stir at a constant speed at 40 °C for 8 h, centrifuge to retain the precipitate, wash with 15 mL of ethanol, and centrifuge at 6000 rpm for 10 min to retain the precipitate, repeat this 3 times, the precipitate obtained is amino-functionalized mesoporous silica nanoparticles (MSN-NH_2_).

### Preparation of amino-functionalized mesoporous silica nanoparticles with targeted peptide (MSN-NH_2_-MG1) modifications

50 mg of aminated mesoporous silica nanoparticles were uniformly dispersed in DMF by ultrasonication and stirring, and 20 mg of targeting peptide was added. Then the condensation agent HBTU and pH adjuster DIEA were added and reacted overnight. After the reaction, the supernatant was removed by centrifugation and the unreacted target peptide was washed with DMF, and the product collected by vacuum drying overnight was the aminated mesoporous silica nanoparticles (MSN-NH_2_-MG1) with the modified target peptide.

### Preparation of nanoparticles loaded with miR-26a-5p

The amino-mesoporous silica nanoparticles modified with the targeting peptide were sterilized and ultrasonically dispersed in DEPC water, and microRNA-26a-5p was dissolved in DEPC water, and the above two liquids were mixed and ultrasonicated for 30 min and then placed in a shaker overnight, and the nanoparticles were loaded with miR-26a-5p through the electrostatic effect of positive and negative charges. The prepared nanoparticle suspensions were put into -20 ℃ refrigerator and frozen for further use.

### Standard curve determination of targeting peptide and miR-26a-5p

The targeting peptide and miR-26a-5p were dissolved in deionized water and DEPC water, respectively, and diluted in a certain ratio to prepare different concentrations of standard solutions containing the targeting peptide or microRNA. The absorbance of each concentration of target peptide or miR-26a-5p was measured by UV–Vis spectrophotometer, and the absorbance-concentration curve was established and linearly fitted.

### Particle size analysis and zeta potential analysis of nanoparticles

The above-prepared MSN, MSN-NH_2_, MSN-NH_2_-MG1 and MSN-NH_2_-MG1@miR nanoparticles were uniformly dispersed into deionized water by sonication for 2 h. Then the nanoparticle suspensions were aspirated into a quartz cuvette and the particle size distribution of the nanoparticles was determined by dynamic light scattering technique of laser particle size measurement.

For the potential determination of nanoparticles, the nanoparticle suspension was first added to the quartz cuvette, and the target electrode was inserted and connected to the sensor. The zeta potential of the nanoparticles is also determined by the electrophoretic light scattering technique of the laser particle sizer.

### Transmission electron microscopy characterization of nanoparticles

MSN, MSN-NH_2_, MSN-NH_2_-MG1 and MSN-NH_2_-MG1@miR nanoparticles were dispersed in deionized water and ultrasonically disperse well. The nanoparticle suspension was added dropwise on a copper mesh (200–300 mesh/inch), and after the liquid dried, it was put into TEM to observe the size of the nanoparticles.

### Fourier transform infrared (FTIR) spectroscopic characterization of nanoparticles

To verify whether the nanoparticles were chemically modified successfully, FTIR was used to characterize MSN, MSN-NH_2_, and MSN-NH_2_-MG1 nanoparticles. The nanoparticles and spectral grade potassium bromide were mixed well, dried by IR and pressed, and tested using an IR spectrometer to measure air as background noise reduction.

### Thermogravimetric analysis of nanoparticles

A certain mass of nanoparticles is placed in a small crucible of the thermogravimetric analyzer and heated in the range of 30–800 ℃ with a heating rate of 10 ℃/min, and a nitrogen atmosphere is maintained throughout. The real-time residual weight and temperature of the sample were recorded, and the degree of modification of the nanoparticles was determined by the residual weight percentage of nanoparticles-temperature graph.

### In vitro release study of miRNA-26a-5p

To investigate the slow release of microRNA by nano-delivery system, an in vitro release behavior study of MSN-NH2-MG1@miR nanoparticles was conducted. The miR-loaded nanoparticles were placed in DEPC water, and 10 μl of liquid was pipetted into the microdetector every 12 h for absorbance testing, and the miR concentration in DEPC water was calculated from its standard curve to determine the degree of miR release.

### In vitro cytocompatibility study of nanoparticles

Live-dead staining of human umbilical vein endothelial cells: HUVECs of logarithmic growth phase were grown into 24-well plates, and after the cells were adhered to the wall overnight, the medium was discarded and the culture medium containing nanoparticles was switched. The medium was aspirated on days 1 and 3, respectively, and the cells were washed with PBS before adding the cell viability fluorescent dye Calcein-AM/PI. After incubation for 15 min, the well plates were placed under an inverted fluorescent microscope to observe cell survival.

### Nanoparticle cell proliferation toxicity test

The logarithmic growth phase HUVECs were seeded onto 96-well plates, and after the cells were plastered, they were switched to culture medium containing nanoparticles, and the medium was changed every 24 h with fresh medium. CCK-8 staining solution was added to the medium of 96-well plates on days 1, 2 and 3, and the absorbance at 450 nm was measured by an enzyme marker after incubation for 1 h in the incubator. The effect of nanoparticles on cell proliferation was determined by comparing the difference in absorbance between the experimental and control groups.

### Animal and pain models

Male C57/BL6 mice (8 weeks old, 22 ± 2 g) were obtained from the central animal facility of Southern Medical University (Guangzhou, China). All the animal studies were carried out according to the approved protocols and guidelines of the Institutional Animal Ethical Care Committee of Southern Medical University Experimental Animal Centre and the International Association for the Study of Pain. The animals were housed under standard conditions of light and dark cycles (12 h:12 h, temperature 25℃) with free access to food and water. They were allowed to acclimate to these conditions for at least 3 days before all experiments. The spared nerve injury (SNI) model was performed as previously described [72]. Briefly, after mice were anesthetized with sevoflurane (2–5%), the sciatic nerve near the thigh region were isolated and its branch, the sural, common peroneal and tibial nerves were exposed and separated. The common peroneal and tibial nerves were tightly ligated with 4–0 silk at the trifurcation and then were cut at the distal of silk knot, followed by removing 3–5 mm of the distal nerves end. The sural nerve was carefully leaving intact during the surgery. For mice in sham group, the sciatic nerve was isolated and exposed in the same way only without ligated and cut its branches. Inflammatory pain was established in mice by subcutaneous injection of CFA (20 μl, Sigma, St. Louis, MO) to the left plantar after mice were anesthetized with sevoflurane (2–5%). For mice in sham group, 20 μl of normal saline was subcutaneously injected into the left paw. The mouse PIPN (Paclitaxel-induced peripheral neuropathy) model was established according to previously described methods [73–75]. Briefly, paclitaxel (ACMEC Biochemical, Shanghai, China) was injected intraperitoneally (i.p.) at a dose of 5 mg/kg for 5 consecutive days. Control animals received the same volume of sterile saline. All the animal studies were carried out according to the approved protocols and guidelines of the Institutional Animal Ethical Care Committee of Southern Medical University Experimental Animal Centre and the International Association for the Study of Pain [76].

### Drugs and reagents

Complete Freund’s adjuvant (CFA) (#F5881) was purchased from Sigma-Aldrich. Paclitaxel (P36050-50 mg) was purchased from ACMEC Biochemical (Shanghai, China). Foxy5 (#S6961) was purchased from Selleck. Foxy5 was dissolved in DMSO at a concentration of 1 mg/mL. mmu-miR-26a-5p agomir, agomir-NC were all purchased from GenePharma Co. Ltd. (Shanghai, China). The mmu-miR-26a-5p agomir dissolved concentration was 200 nmol/mL in DEPC-treated Water.

### Intrathecal injections

For intrathecal injection, the method described previously was used [77]. Mice were anesthetized with isoflurane. The thumb and middle finger of the left hand firmly old the paralumbar region of the iliac crest, and the index finger is placed on the tip of the spinous process of the sixth lumbar vertebra (L6), the highest point of the vertebral body. All intrathecal injections were delivered in a total volume of 10 μl. The needle is inserted into the fifth intervertebral space (L5–L6), resulting in a sudden lateral movement of the tail. The needle is held in place for 10 s and then slowly withdrawn to avoid spillage.

### Vonfrey test

Mice were acclimated to the test environment for 30 min before baseline nociceptive threshold testing. Mechanical thresholds were measured using the von Frey monofilament (Semmes Weinstein) "up-down" method. Mice were individually placed in glass boxes (9 × 25 × 25 cm) on grid iron racks. During the test, the von Frey filament was applied to the plantar surface of the hind paw, bending and holding the filament for 3 s. The threshold force causing hind paw retraction was recorded, with measurements repeated at a minimum interval of 20 min for averaging. Researchers conducting the tests were blinded to the treatment groups to prevent bias.

### Hargreaves test

Thermal hyperalgesia was evaluated by following a thermal stimulus paradigm adapted from published reports [78]. Infrared heat was applied to the plantar surface of the hind paw using a Hargreaves device (Ugo Basile). The time taken for the mice to withdraw their paw (thermal latency) was measured, with a cutoff latency of 20 s. This test was repeated three times at 20-min intervals for each mouse, and the average thermal latency was calculated.

### Open field test

The apparatus consisted of a large area composed of plastic, surrounded by walls that were 100 cm high. The floor was 50 × 50 cm for mice; the overall illumination was 100–200 lx. Each animal was gently placed in the centre of the open field, and its behaviour was videotaped. The time in the centre (mice, 25 × 25 cm) and the distance the animals travelled were measured using ANY-maze (Stoelting Co., IL, USA).

### Western blotting

Mouse spinal cord L4-5 segment tissues were digested in RIPA extraction buffer (Beyotime, China). Protein samples were separated by 10% SDS-PAGE and transferred onto PVDF (polyvinylidene difluoride) membranes (Millipore, United States) in tank transfer system (Bio-Rad, United States). Membranes were blocked with 5% non-fat milk in buffer containing 0.1% Tween-20 (TBST) for 1 h, washed three times in TBST, and incubated overnight at 4℃ with primary antibodies including rabbit anti-Wnt5a (1:1000 dilution; Proteintech; USA; 55184-1-AP), mouse ROR2 Monoclonal antibody(1:1000 dilution; Proteintech; USA; 67906-1-Ig), mouse JNK Monoclonal antibody (1:1000 dilution; Proteintech; USA; 66210-1-Ig), rabbit Phospho-JNK Recombinant antibody (1:1000 dilution; Proteintech; USA; 80024-1-RR), rabbit anti-iNOS (1:1000 dilution; Proteintech; US; 18985-1-AP), rabbit anti-CD206(1:1000 dilution; Proteintech; US; 18704-1-AP), mouse Beta Actin Monoclonal antibody (1:10000 dilution; Proteintech; USA; 66009-1-Ig). After incubation with the HRP-conjugated goat anti-rabbit IgG secondary antibody (1:10,000 dilution, Da-UN, China), immunoreactive bands were detected by enhanced chemiluminescence (Millipore, United States). The protein bands were quantitatively analysed using ImageJ software 1.8.0 (National Institutes of Health, Bethesda, MA, USA).

### Cell culture and treatment

BV2 cell line was obtained from Gaining Biological Technology Co., Ltd (Shanghai, China). Immortalized mouse BV2 microglial cell lines were cultured in DMEM medium with 10% FBS and 1% penicillin/streptomycin at 37 °C in a humidified atmosphere containing 95% air and 5% CO2, and the medium was changed daily. The cells were digested with 0.25% trypsin when they reached 70% confluence and subcultured for further passages. BV2 cells were treated with or without LPS (1 μg/mL) and Foxy5 (1 μg/mL) for 24 h.

### ELISA for the determination of cytokine levels

Spinal cord L4-5 of mice were lysed after different treatments, and cytokine levels were determined. The concentrations of IL-1β, TNF-α, IL-6 and IL-10 levels were measured by ELISA according to the manufacturer’s instructions (Proteintech, USA). Optical density (OD) was measured at 450 nm using a microplate reader (Thermo Scientific).

### Immunofluorescence staining

L4-5 spinal cord samples were removed and cut into 20 μm frozen cryosections using a microtome. Tissue sections were fixed for 10 min in 4% paraformaldehyde (Solarbio, China) at room temperature, then permeabilized and blocked with 0.5% TritonX-100 (Sigma-Aldrich) and 3% bovine serum albumin (BSA, Solarbio, China) at room temperature for 1 h. Next, spinal cord tissue sections were incubated with diluted primary antibodies against Iba1 (Wako 559-24761), GFAP (Proteintech 16825-1-AP), MAP2 (Proteintech 17490-1-AP). Sections were then incubated with appropriate secondary antibodies (1:500, Alexa Fluor 488-labeled goat anti-rabbit, mouse IgG, Jackson Immuno Research, West Grove, PA) for 1 h at room temperature. Finally, the slides were mounted with anti-quenching DAPI (49,6-diamino-2-phenylindole) fluorescent mounting medium. Images were acquired with a confocal laser scanning microscope system (Zeiss, LSM 980, Germany) and upright manual fluorescence microscope (Zeiss, ImagerD2, Germany), then processed with Adobe Photoshop 8.0 software (Adobe Systems, Mountain View, CA). Grayscale conversion analysis of fluorescence images of IBA1 was performed with ImageJ software 1.8.0 (National Institutes of Health, Bethesda, MA, USA).

### Statistical analysis

Statistical analyses were performed using SPSS 22.0 Statistics (IBM SPSS Statistics for Version 22.0, IBM Corp, North Castle, NY, USA). All data are expressed as mean ± SEM. For data obtained via behavioral test data, two-way ANOVA with repeated measures followed by Tukey’s post hoc test was used to analyze the differences between different groups. For data obtained via qRT-PCR, Western blotting, Elisa and immunofluorescence staining, one-way ANOVA followed by Tukey’s post hoc test was used for multiple group comparisons. Shapiro–Wilk normality test indicated that the data have normal distribution; therefore, comparisons were done using parametric tests. Differences were statistically significant when P value less than 0.05.

### Supplementary Information

**Additional file 1:** **Figure S1. A** Concentration of nanoparticles stored for 9 and 12 months. **B** Release rate in ACSF between 0 and 12 months’ nanoparticles. **Figure S2. A** HE staining of liver and kidney. Scale bars are 100 μm. **B** The ALT, AST, BUN and Cr levels, respectively. **Figure S3. (A–F)** 50% paw withdraw threshold (PWT) of left hind paw of different treatment groups mice at day 8, 10, 28, 35, 42, 49 in SNI model. Data are represented as mean ± sem. *p < 0.05, **p < 0.01, ***p < 0.001. **Figure S4. A** Total distance in open field test showed no difference in mouse motor function between groups.** B** Rotarod test showed no difference in mouse motor function between groups. **Figure S5.** Immunofluorescent study revealed that the enrichment of nanoparticles (Cy5 red) in microglia (IBA1 green) in the L4–5 spinal dorsal horn of SNI mice, ipsilateral and contralateral. **Figure S6. A** Immunofluorescence studies revealed that nanoparticles (Cy5 red) are rarely found in astrocytes in the dorsal horn of the L4-5 spinal cord of SNI mice. The blue spots are DAPI nuclear staining (Scale bar: 50 μm).** B** Quantification showing the number of microglia cells containing nanoparticle was significantly increased in the miR@MSN-peptide group compared with miR@MSN group. Data are represented as mean ± sem. *p < 0.05, **p < 0.01, ***p < 0.001. **Figure S7.** Immunofluorescent study revealed that the enrichment of nanoparticles (Cy5 red) in DRG. The blue spots are DAPI nuclear staining (Scale bar: 50 μm), Green: NeuN^+^. **Figure S8.** Quantification showing the number of SCDH activated microglia cells in different group at POD 21. Data are represented as mean ± sem. *p < 0.05, **p < 0.01, ***p < 0.001. **Figure S9. (A, B)** 50% paw withdraw threshold (PWT) of left hind paw of different treatment groups mice at day 1, 3 in CFA model. **(C, D)** Thermal latency(s) of left hind paw of mice in different treatment inflammatory pain mice groups. Data are represented as mean ± sem. *p < 0.05, **p < 0.01, ***p < 0.001. **Figure S10. (A–C)** 50% paw withdraw threshold (PWT) of left hind paw of different treatment CIPN mice groups. Data are represented as mean ± sem. *p < 0.05, **p < 0.01, ***p < 0.001.

## Data Availability

The data that support the findings of this study are available from the corresponding author upon reasonable request.

## Abbreviations

ANOVA: Analysis of variance
CaMKII: Calmodulin-dependent protein kinase 2
ARA290: An erythropoietin-derived polypeptide
CFA: Complete Freund's adjuvant
Contra: Contralateral
DAPI: 4′,6-Diamidino-2-Phenylindole
DEPC: Diethypyrocarbonate
ELISA: Enzyme-linked immunosorbent assay
FITC: Fluorescein isothiocyanate
GO: Gene ontology
GFAP: Glial fibrillary acidic protein
hPMSCs: Human placenta-derived mesenchymal stem cells
Iba1: Ionized calcium-binding adapter molecule 1
i.t.: Intrathecal injection
IL-1β: Interleukin-1β
IL-6: Interleukin-6
Ipsi: Ipsilateral
MSN: Mesoporous silica nanoparticles
miRNA: MicroRNA
NC: Negative control
NFAT: Nuclear factor of activated T cells 1
qRT-PCR: Quantitative real-time polymerase chain reaction
QX-314: Membrane-impermeable Na^+^-channels blocker
SNI: Spared nerve injury
SEM: Standard error of the mean
TBST: Tris-buffered saline and Tween
TNF-α: Tumor necrosis factor-α
Wnt5a: Wingless-Type MMTV Integration Site Family, Member 5A
Δ9-THC: Δ9-Tetrahydrocannabinol, a cannabinoid
