# Supplementary material for: A novel microglia-targeting strategy based on nanoparticle-mediated delivery of miR-26a-5p for long-lasting analgesia in chronic pain
Source: J Nanobiotechnology. 2024 Mar 23;22:128. doi: 10.1186/s12951-024-02420-9 (PMC10960380; doi:10.1186/s12951-024-02420-9)
Supplement: Supplementary file 1 — Additional file 1: Figure S1. A Concentration of nanoparticles stored for 9 and 12 months. B Release rate in ACSF between 0 and 12 months’ nanoparticles. Figure S2. A HE staining of liver and kidney. Scale bars are 100 μm. B The ALT, AST, BUN and Cr levels, respectively. Figure S3. (A–F) 50% paw withdraw threshold (PWT) of left hind paw of different treatment groups mice at day 8, 10, 28, 35, 42, 49 in SNI model. Data are represented as mean ± sem. *p < 0.05, **p < 0.01, ***p < 0.001. Figure S4. A Total distance in open field test showed no difference in mouse motor function between groups. B Rotarod test showed no difference in mouse motor function between groups. Figure S5. Immunofluorescent study revealed that the enrichment of nanoparticles (Cy5 red) in microglia (IBA1 green) in the L4–5 spinal dorsal horn of SNI mice, ipsilateral and contralateral. Figure S6. A Immunofluorescence studies revealed that nanoparticles (Cy5 red) are rarely found in astrocytes in the dorsal horn of the L4-5 spinal cord of SNI mice. The blue spots are DAPI nuclear staining (Scale bar: 50 μm). B Quantification showing the number of microglia cells containing nanoparticle was significantly increased in the miR@MSN-peptide group compared with miR@MSN group. Data are represented as mean ± sem. *p < 0.05, **p < 0.01, ***p < 0.001. Figure S7. Immunofluorescent study revealed that the enrichment of nanoparticles (Cy5 red) in DRG. The blue spots are DAPI nuclear staining (Scale bar: 50 μm), Green: NeuN+. Figure S8. Quantification showing the number of SCDH activated microglia cells in different group at POD 21. Data are represented as mean ± sem. *p < 0.05, **p < 0.01, ***p < 0.001. Figure S9. (A, B) 50% paw withdraw threshold (PWT) of left hind paw of different treatment groups mice at day 1, 3 in CFA model. (C, D) Thermal latency(s) of left hind paw of mice in different treatment inflammatory pain mice groups. Data are represented as mean ± sem. *p < 0.05, **p < 0.01, ***p < 0.001. Figure [file 12951_2024_2420_MOESM1_ESM.docx]

**A novel microglia-targeting strategy based on nanoparticle-mediated delivery of miR-26a-5p for long-lasting analgesia in chronic pain**

**Yitian Lu^1, 3, 5†^, Shuai Liu^2†^, Peng Wang^3^, Xiangna Guo^3^, Zaisheng Qin^3^, Honghao Hou^2^*, and Tao Tao^1, 4^***

^1^Department of Anesthesiology, Zhujiang hospital, Southern Medical University, Guangzhou, Guangdong, People’s Republic of China.

^2^Guangdong Provincial Key Laboratory of Construction and Detection in Tissue Engineering, School of Basic Medical Sciences, Southern Medical University, Guangzhou, Guangdong, People’s Republic of China.

^3^Department of Anesthesiology, Nanfang hospital, Southern Medical University, Guangzhou, Guangdong, People’s Republic of China.

^4^Department of Anesthesiology, Central People's Hospital of Zhanjiang, Zhanjiang, Guangdong, China.

^5^Neuroscience Research Institute and Department of Neurobiology, School of Basic Medical Sciences, Key Laboratory for Neuroscience, Ministry of Education/National Health Commission, National Health Commission and State Key Laboratory of Natural and Biomimetic Drugs, Peking University, Beijing, China.

* E-mail: [taotaomzk@smu.edu.cn](mailto:taotaomzk@smu.edu.cn) (T. Tao); ss.hhh89@hotmail.com(H.H. Hou)

^†^Yitian Lu and Shuai Liu contributed equally to this work.


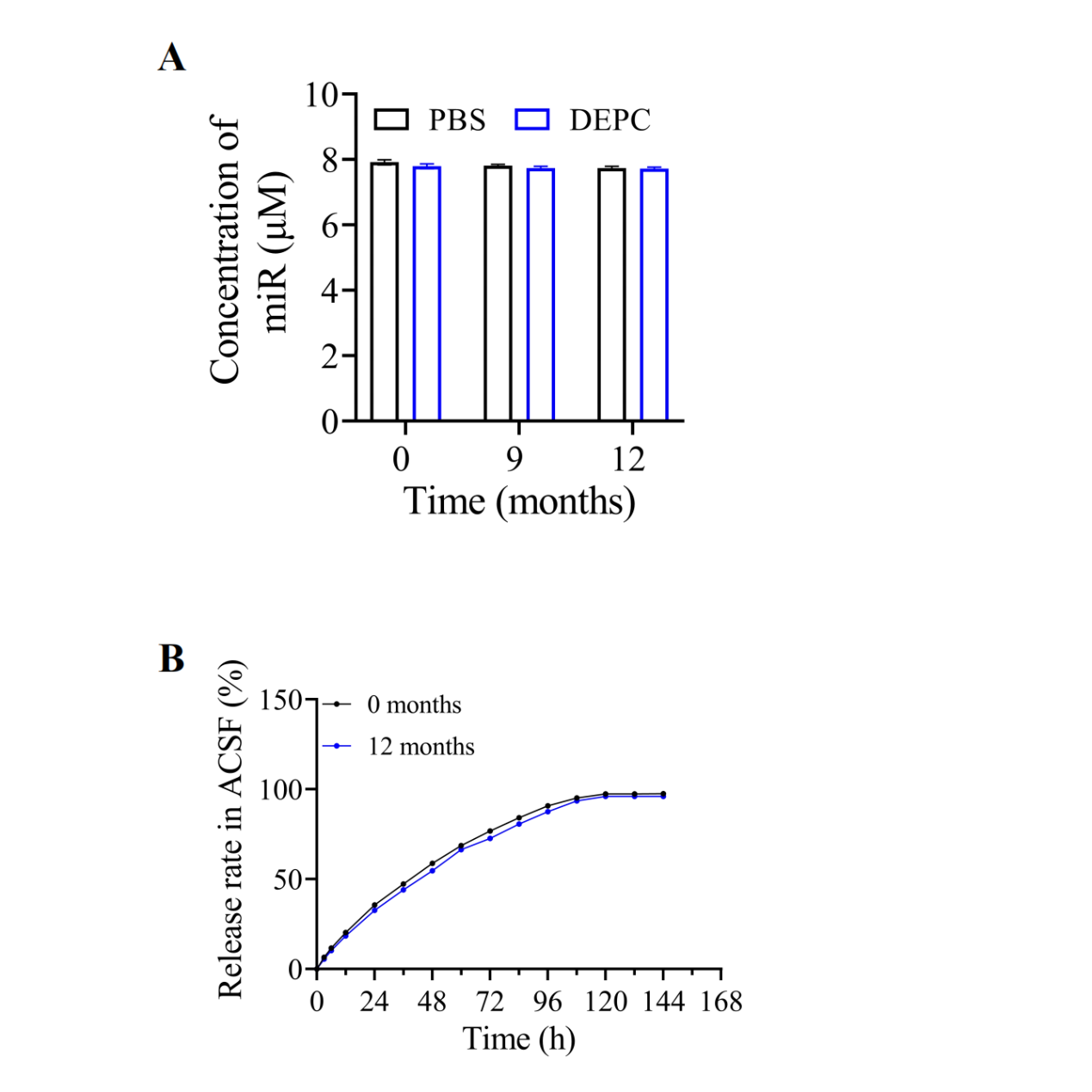


**Figure S1 A** Concentration of nanoparticles stored for 9 and 12 months. **B** Release rate in ACSF between 0 and 12 months’ nanoparticles.

**
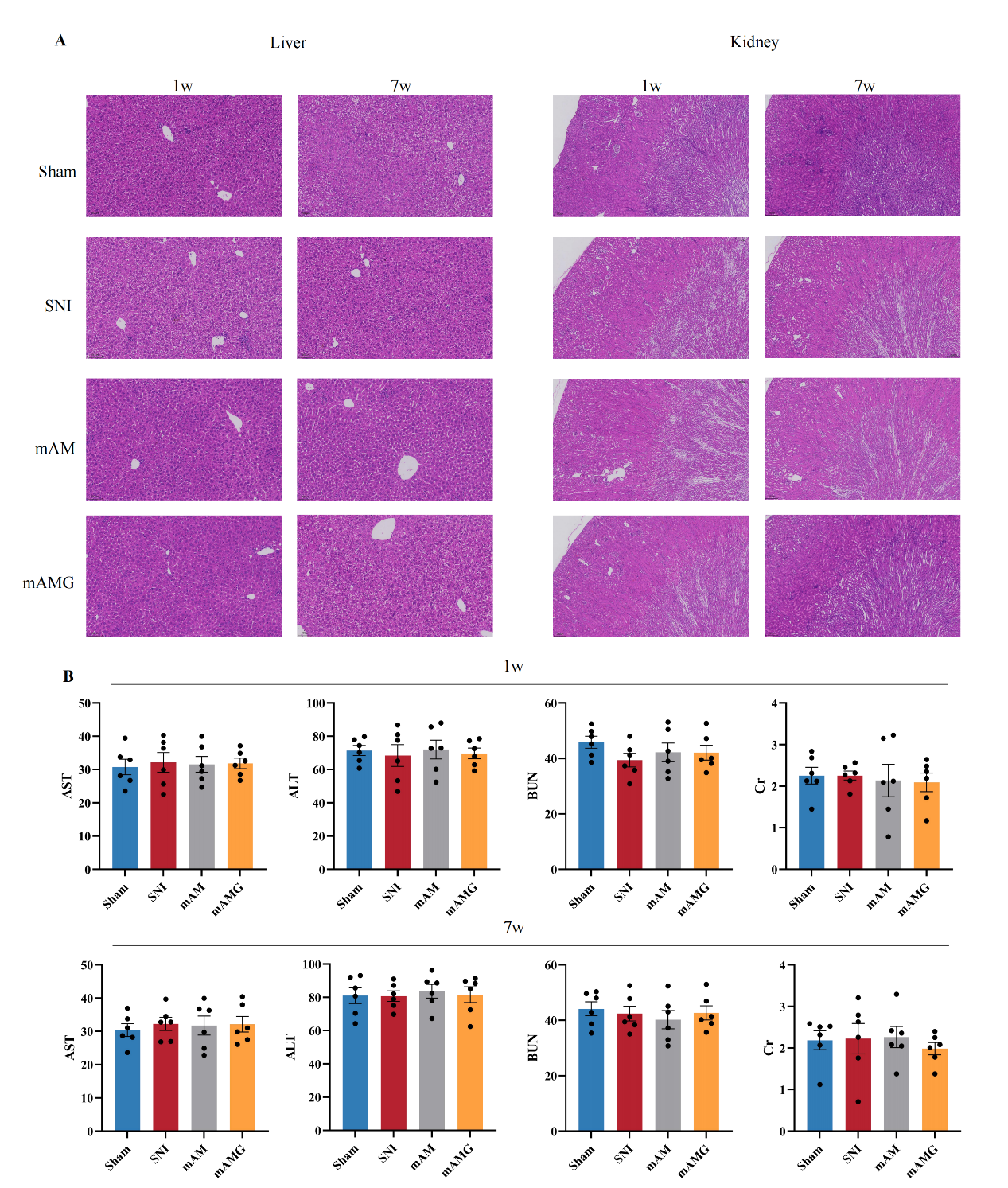
**


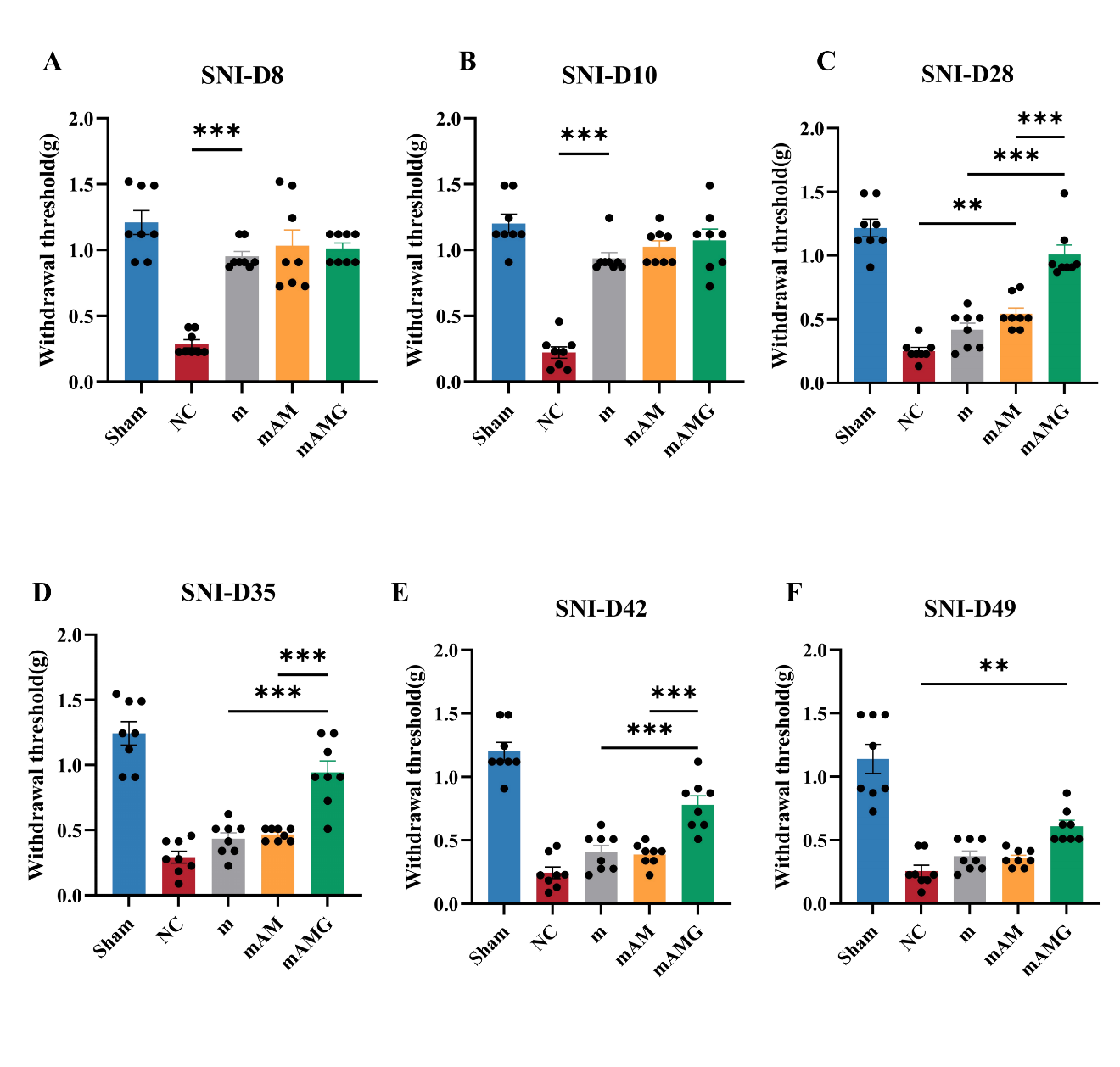


**Figure S3 (A-F)** 50% paw withdraw threshold (PWT) of left hind paw of different treatment groups mice at day 8, 10, 28, 35, 42, 49 in SNI model. Data are represented as mean ± sem. *p< 0.05, **p< 0.01, ***p< 0.001.

**
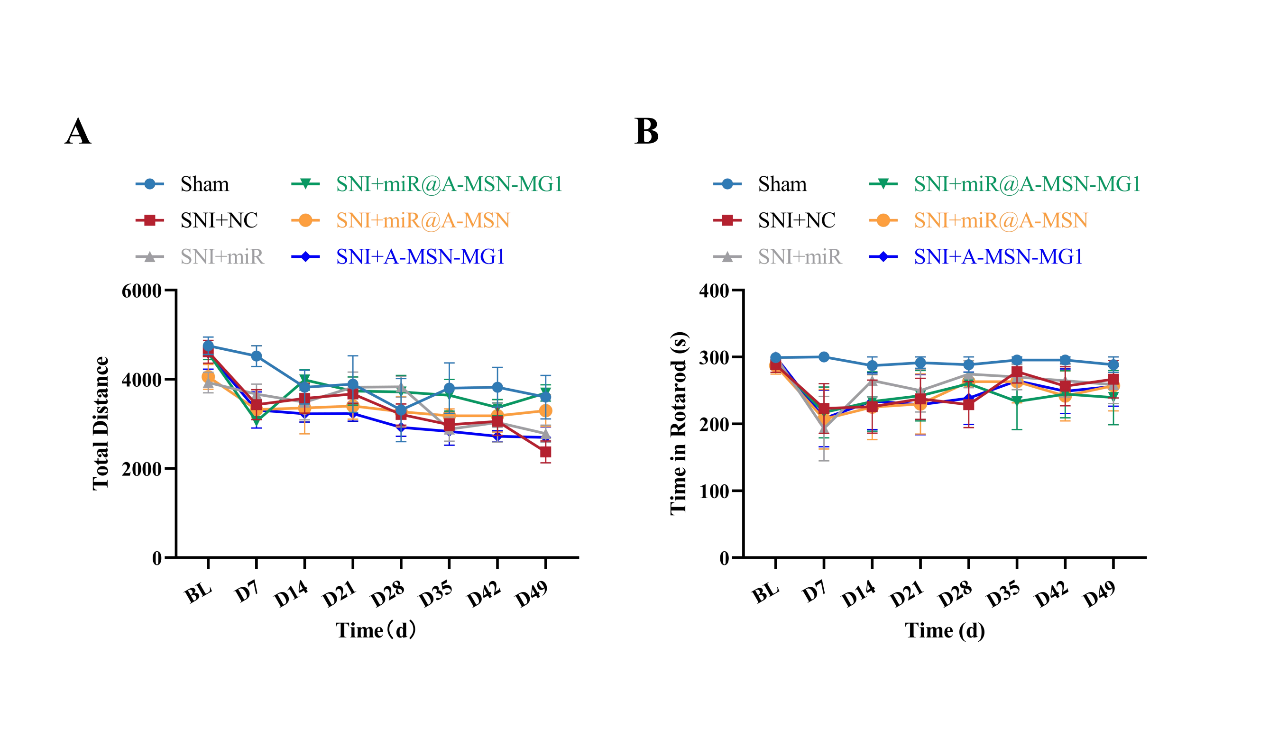
 Figure S4 A** Total distance in open field test showed no difference in mouse motor function between groups. **B** Rotarod test showed no difference in mouse motor function between groups.


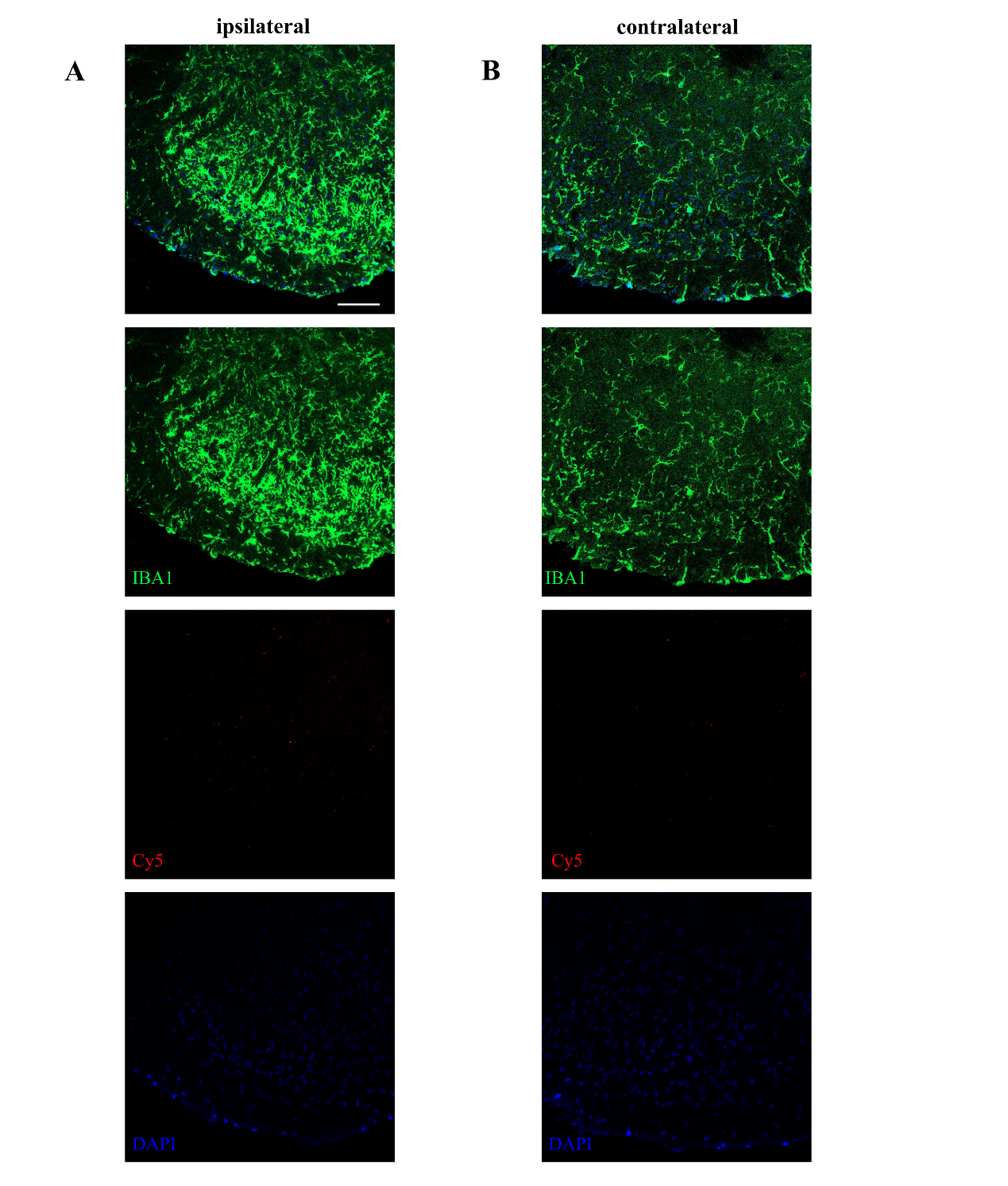
 **Figure S5** Immunofluorescent study revealed that the enrichment of nanoparticles (Cy5 red) in microglia (IBA1 green) in the L4–5 spinal dorsal horn of SNI mice, ipsilateral and contralateral.


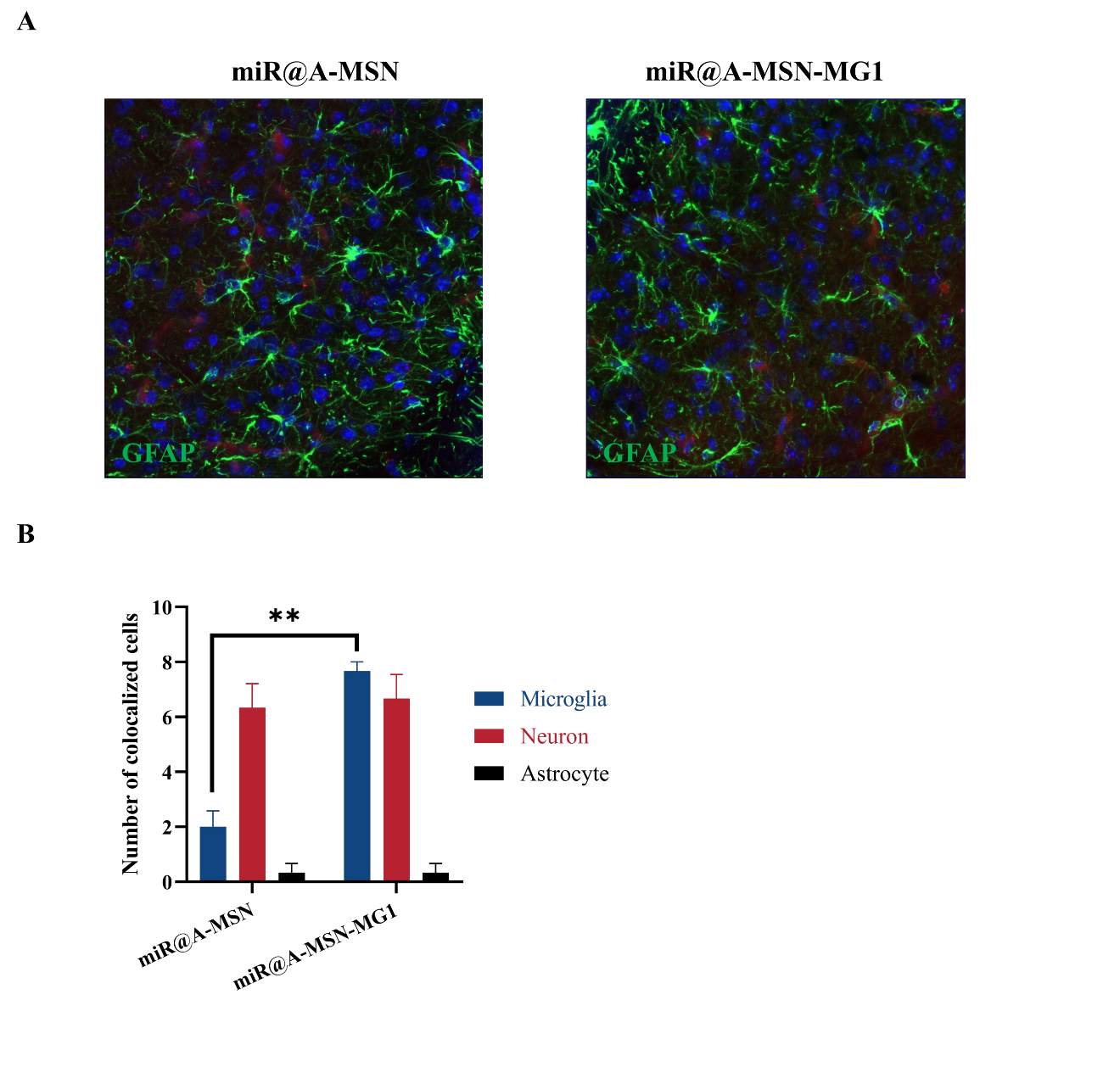


**Figure S6 A** Immunofluorescence studies revealed that nanoparticles (Cy5 red) are rarely found in astrocytes in the dorsal horn of the L4-5 spinal cord of SNI mice. The blue spots are DAPI nuclear staining (Scale bar: 50 μm). **B** Quantification showing the number of microglia cells containing nanoparticle was significantly increased in the miR@MSN-peptide group compared with miR@MSN group. Data are represented as mean ± sem. *p< 0.05, **p< 0.01, ***p< 0.001.

**
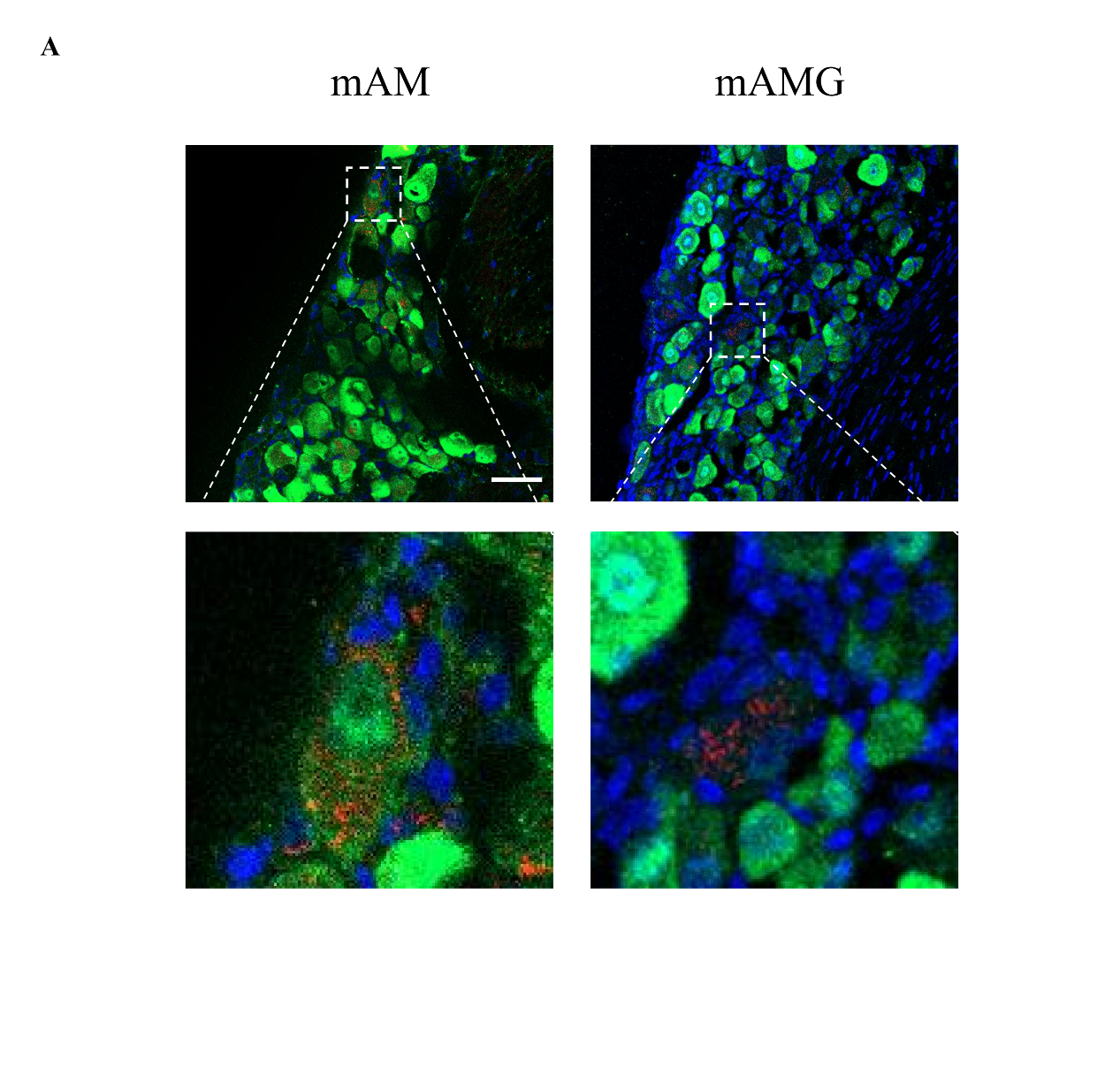
 Figure S7** Immunofluorescent study revealed that the enrichment of nanoparticles (Cy5 red) in DRG. The blue spots are DAPI nuclear staining (Scale bar: 50 μm), Green: NeuN^+^.


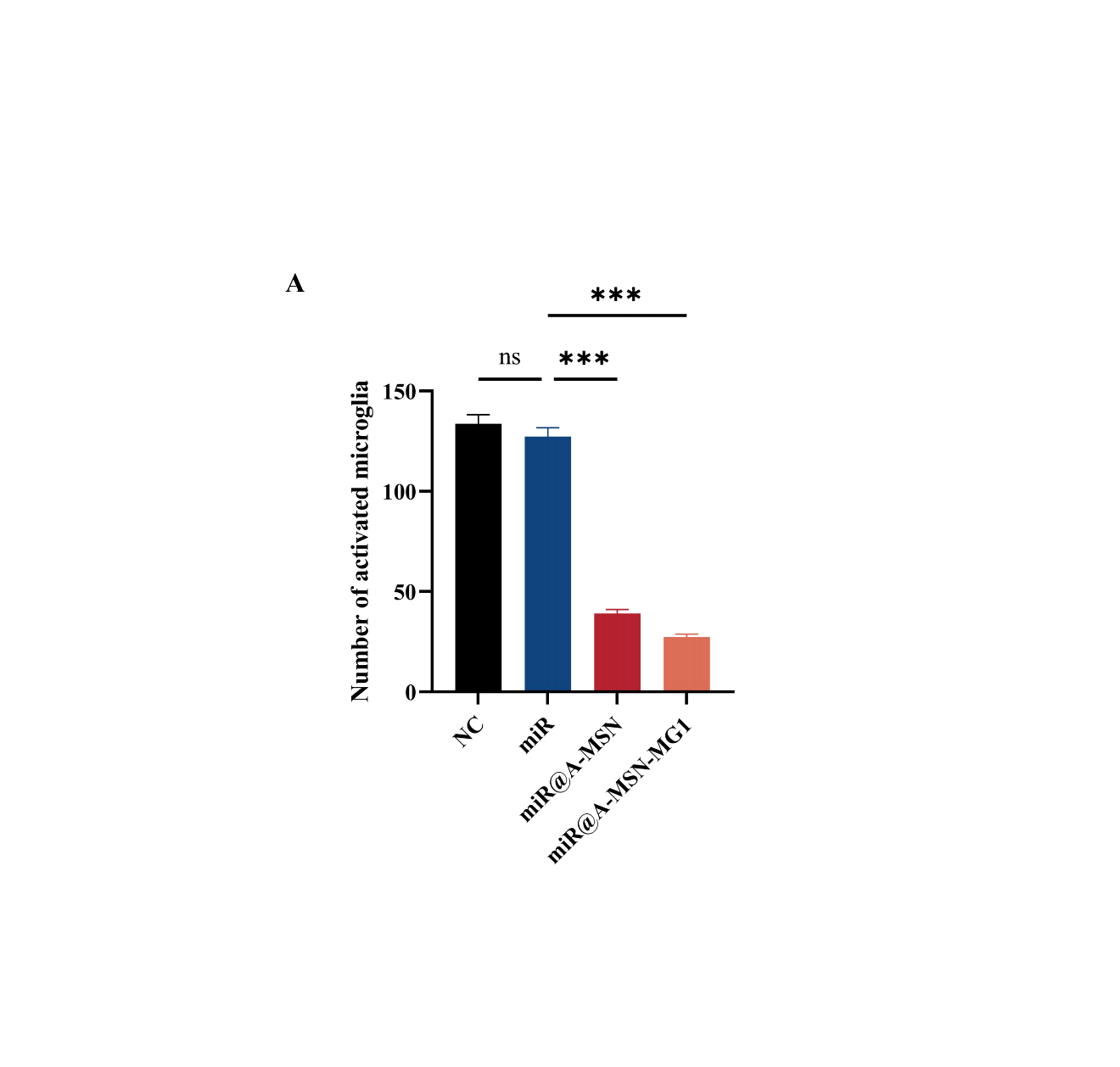


**Figure S8** Quantification showing the number of SCDH activated microglia cells in different group at POD 21. Data are represented as mean ± sem. *p< 0.05, **p< 0.01, ***p< 0.001.


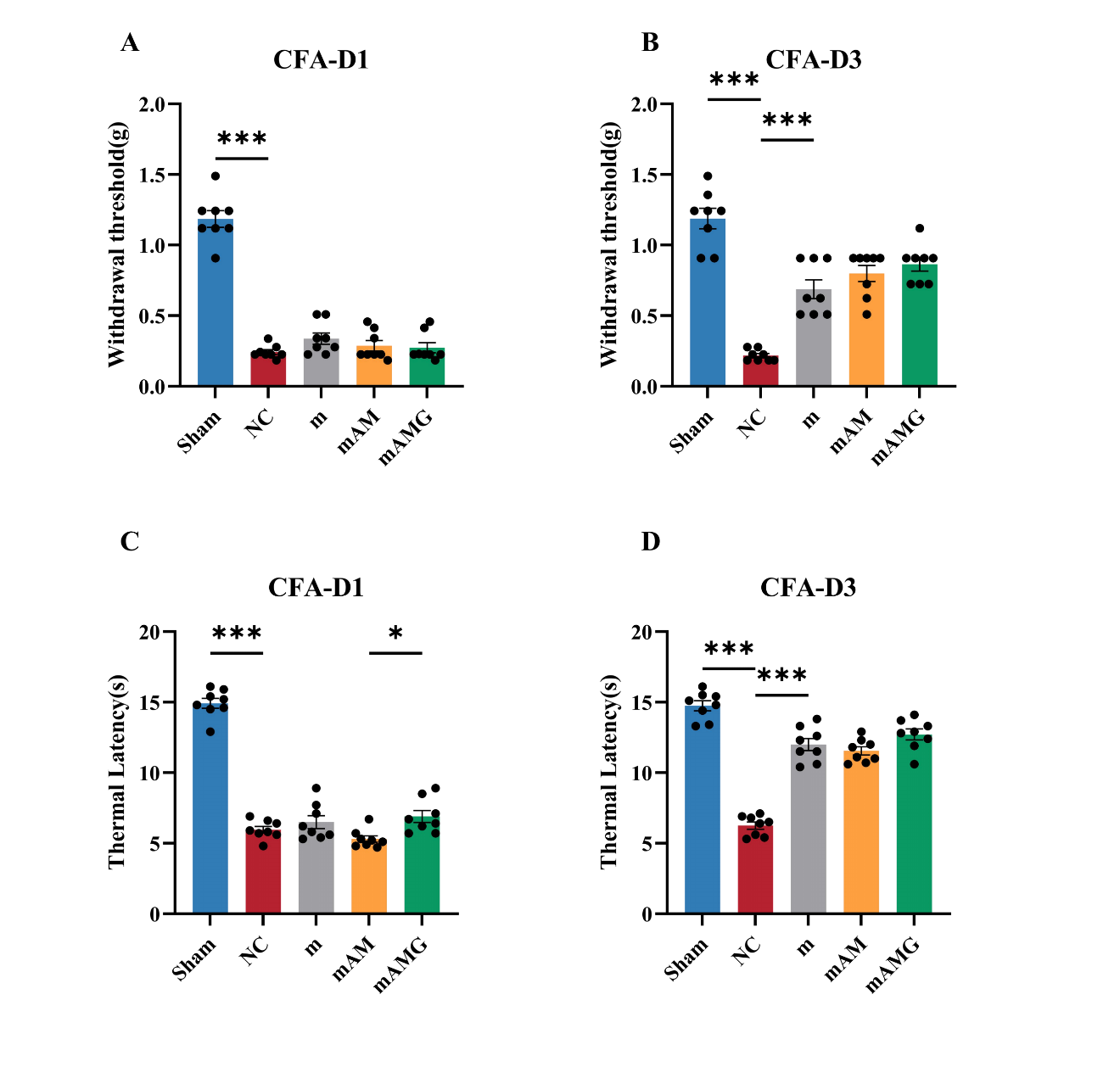


**Figure S9 (A, B)** 50% paw withdraw threshold (PWT) of left hind paw of different treatment groups mice at day 1, 3 in CFA model. **(C, D)** Thermal latency(s) of left hind paw of mice in different treatment inflammatory pain mice groups. Data are represented as mean ± sem. *p< 0.05, **p< 0.01, ***p< 0.001.


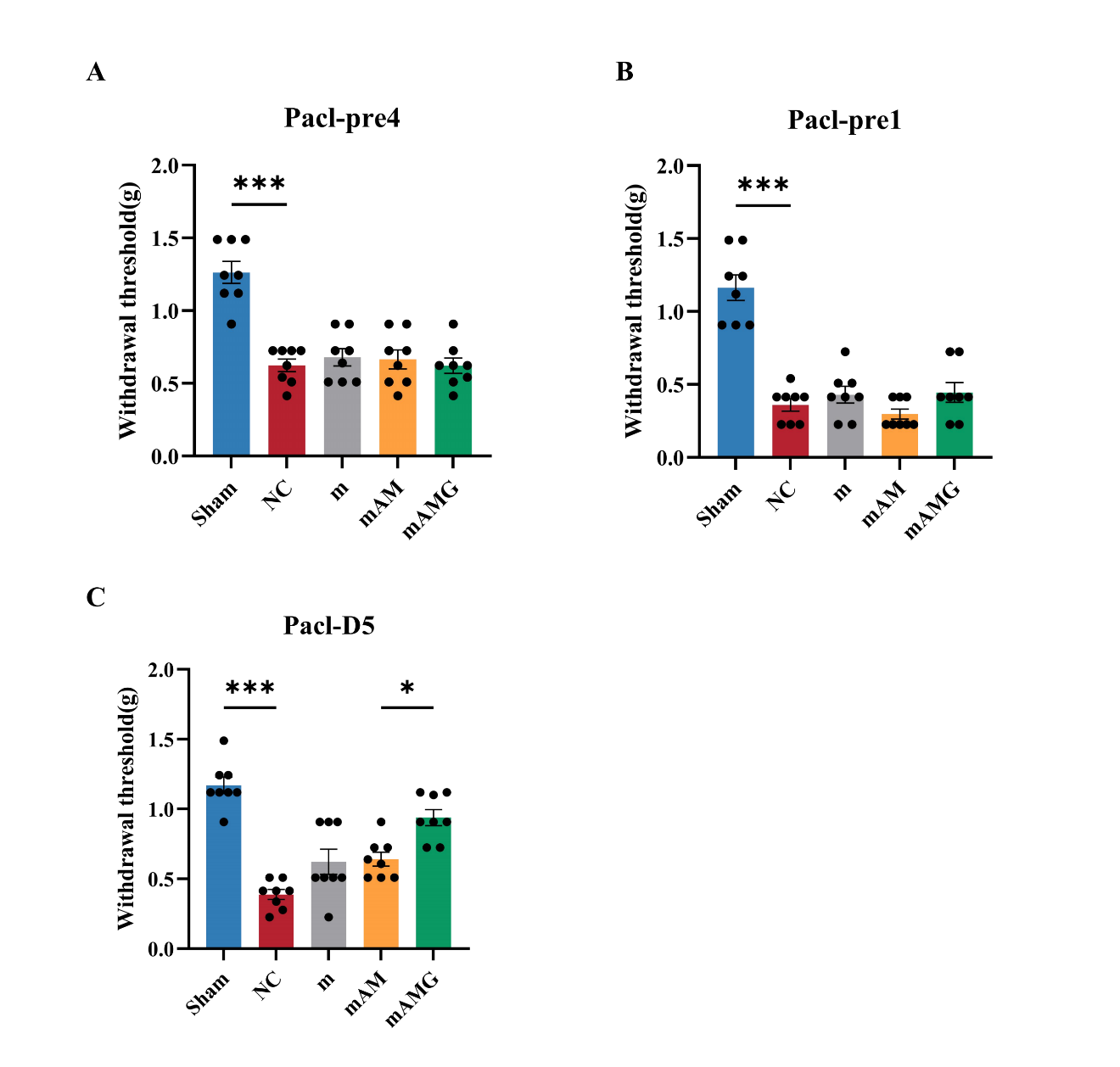


**Figure S10 (A-C)** 50% paw withdraw threshold (PWT) of left hind paw of different treatment CIPN mice groups. Data are represented as mean ± sem. *p< 0.05, **p< 0.01, ***p< 0.001.
